# Anaplerotic Triheptanoin Diet Enhances Mitochondrial Substrate Use to Remodel the Metabolome and Improve Lifespan, Motor Function, and Sociability in MeCP2-Null Mice

**DOI:** 10.1371/journal.pone.0109527

**Published:** 2014-10-09

**Authors:** Min Jung Park, Susan Aja, Qun Li, Alicia L. Degano, Judith Penati, Justin Zhuo, Charles R. Roe, Gabriele V. Ronnett

**Affiliations:** 1 The Center for Metabolism and Obesity Research, The Johns Hopkins University, School of Medicine, Baltimore, MD, United States of America; 2 Department of Neuroscience, The Johns Hopkins University, School of Medicine, Baltimore, MD, United States of America; 3 Department of Neurology, The Johns Hopkins University, School of Medicine, Baltimore, MD, United States of America; 4 Department of Biological Chemistry, The Johns Hopkins University, School of Medicine, Baltimore, MD, United States of America; 5 Departamento de Química Biológica, CIQUIBIC-CONICET, Universidad Nacional de Córdoba, Córdoba, Argentina; 6 Department of Brain Sciences, DGIST, Daegu, South Korea; University of Insubria, Italy

## Abstract

Rett syndrome (RTT) is an autism spectrum disorder (ASD) caused by mutations in the X-linked *MECP2* gene that encodes methyl-CpG binding protein 2 (MeCP2). Symptoms range in severity and include psychomotor disabilities, seizures, ataxia, and intellectual disability. Symptom onset is between 6-18 months of age, a critical period of brain development that is highly energy-dependent. Notably, patients with RTT have evidence of mitochondrial dysfunction, as well as abnormal levels of the adipokines leptin and adiponectin, suggesting overall metabolic imbalance. We hypothesized that one contributor to RTT symptoms is energy deficiency due to defective nutrient substrate utilization by the TCA cycle. This energy deficit would lead to a metabolic imbalance, but would be treatable by providing anaplerotic substrates to the TCA cycle to enhance energy production. We show that dietary therapy with triheptanoin significantly increased longevity and improved motor function and social interaction in male mice hemizygous for *Mecp2* knockout. Anaplerotic therapy in *Mecp2* knockout mice also improved indicators of impaired substrate utilization, decreased adiposity, increased glucose tolerance and insulin sensitivity, decreased serum leptin and insulin, and improved mitochondrial morphology in skeletal muscle. Untargeted metabolomics of liver and skeletal muscle revealed increases in levels of TCA cycle intermediates with triheptanoin diet, as well as normalizations of glucose and fatty acid biochemical pathways consistent with the improved metabolic phenotype in *Mecp2* knockout mice on triheptanoin. These results suggest that an approach using dietary supplementation with anaplerotic substrate is effective in improving symptoms and metabolic health in RTT.

## Introduction

Rett syndrome (RTT, Online Mendelian Inheritance in Man 312750) is an autism spectrum disorder (ASD) that results in severe cognitive and motor disabilities, seizures, and loss of language[1]–[5]. The vast majority of RTT cases are caused by mutations in the X-linked gene for methyl-CpG binding protein 2 (MeCP2), a member of a family of transcriptional factors that binds methyl-CpG DNA base pairs[6], [7], and thus regulates gene expression. RTT affects mainly females, approximately 1 in 10,000 to 15,000 children, accounting for 2–3% of cases of severe mental disability[8]–[10].

There is a growing appreciation that RTT symptoms may be due in part to defects in mitochondrial function[11]. Mitochondria convert nutrients into usable energy through cellular respiration, and typically produce most cellular ATP. Compromised mitochondrial function has been linked to metabolic diseases, neurodegenerative disorders, sarcopenia, and aging[12], [13]. Mitochondria in skeletal muscle from RTT patients have shown abnormal morphology[14]–[18], as well as decreased levels of mitochondrial enzymes and components of the electron transport chain (ETC)[14], [15]. RTT patients are also reported to have elevated serum levels of leptin and decreased adiponectin[19], [20], metabolic read-outs from adipose tissue suggestive of overall body metabolic imbalance. RTT symptoms of psychomotor abnormalities are modeled in mice with knock-outs of the *Mecp2* gene that result in deficient MeCP2 protein[21], and these models have been shown to have mitochondrial abnormalities as well[22].

Energy deficits due to defects in substrate utilization may exist in RTT and in mouse models of the disease, and may underlie some of the deleterious consequences of MeCP2 deficiency, as symptoms present during highly energy-dependent phases of neuromuscular and neurological development. We hypothesized that providing an anaplerotic diet would improve adverse metabolic and psychomotor abnormalities incurred by MeCP2 deficiency. Anaplerotic strategies involve the administration of alternative substrates capable of replenishing the intermediates of the TCA cycle so that it can receive the acetyl group from acetyl coenzyme A (CoA), and provide reducing power (NADH, FADH2) to the ETC to enhance mitochondrial ATP production[23]–[25]. Anaplerotic substrates include pyruvate, glutamine/glutamate, and precursors of propionyl-CoA (odd-chain fatty acids, 5-carbon (C5) ketone bodies, and some amino acids)[26], [27]. An anaplerotic diet, with triheptanoin (C7 triglyceride) providing 30–35% of total caloric intake, has been used successfully to treat some inherited metabolic disorders, the pathology of which could include energy deficiency or redox imbalance resulting from improper TCA cycle function[24], [28]–[31].

We determined whether anaplerotic treatment using dietary triheptanoin could improve health in the setting of MeCP2 deficiency. Starting at 4 weeks of age, male wild type (WT) and *Mecp2* knockout (*Mecp2* KO) mice were fed either triheptanoin diet or an isocaloric control diet containing soybean oil (SBO). The effects of triheptanoin diet were significant. After only two weeks of treatment, there was a clear improvement towards increased insulin sensitivity and glucose tolerance. Chronic treatment with triheptanoin improved motor coordination and social interaction, and increased lifespan. Metabolomics analyses help to delineate the mechanisms of these effects, and show that triheptanoin supplement restores metabolic homeostasis to improve energy availability that would be important for the complex neuromuscular and neurological events that transpire during the period of symptom development in models of RTT. These data have broad implications for ASDs, and demonstrate the value of untargeted metabolomics for assessing metabolic derangements in disease and improvements with therapies.

## Results

### Functional deficits and altered energy homeostasis phenotype in *Mecp2* KO mice

**Figure 1:**
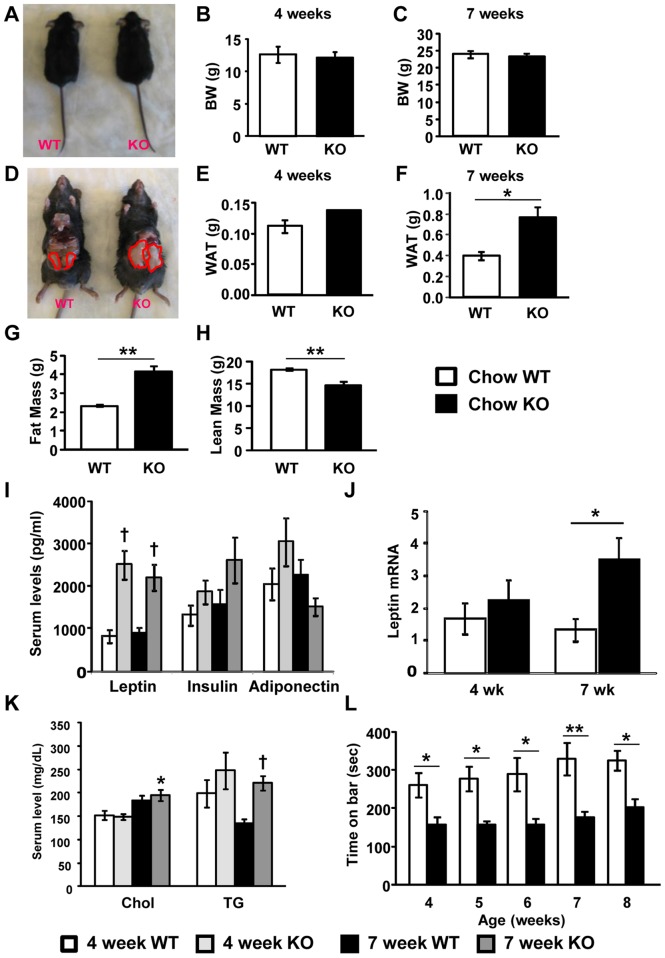
Chow-fed *Mecp2* KO mice have significantly increased WAT mass and serum leptin, and decreased rotarod performance. (A) Representative images of WT and *Mecp2* KO littermates at 7 weeks of age. (B, C) Body weight of WT and *Mecp2* KO mice at 4 weeks (**B**; NS p = 0.2165; unpaired t-test; WT n = 6; KO n =  3) and 7 weeks (**C**, NS p = 0.6845; unpaired t-test; WT n = 4, KO n = 10) of age. (D) Representative images of epididymal WAT from WT and *Mecp2* KO littermates at 7 weeks of age. (E, F) Weights of epididymal WAT of WT and *Mecp2* KO mice at 4 weeks (E; NS p = 0.1834; unpaired t-test; WT n = 6, KO n = 3) and 7 weeks (F, **p = 0.0358; unpaired t-test; WT n = 4, KO n = 10) of age. (G) Body fat mass from WT and KO mice measured by EchoMRI (**p = 0.0003; unpaired t-test; WT n = 4, KO n = 6). (H) Body lean mass from WT and KO mice measured by EchoMRI (**p = 0.0035; unpaired t-test; WT n = 4, KO n = 6). (I) Serum leptin, insulin, and adiponectin levels measured by Multiplex assay at 4 and 7 weeks of age in WT and *Mecp2* KO mice (two-way analysis of variance (ANOVA) with Tukey *post hoc* test; dagger symbols denote differences from matching WT controls, p<0.01; 4-week WT n = 9, 4-week KO n = 8, 7-week WT n = 10, 7-week KO n = 11). (J) mRNA for plasma leptin; data for each group are fold-changes relative to 4-week WT control with actin as a reference gene (two-way analysis of variance (ANOVA) with Tukey *post hoc* test; *p<0.05; 4-week WT n = 9, 4-week KO n = 11, 7-week WT n = 10, 7-week KO n = 8). (K) Serum cholesterol and triglycerides (TG) (two-way analysis of variance (ANOVA) with Tukey *post hoc* test; asterisk denotes difference from 4-week KO, and dagger denotes difference from 7-week WT; 4-week WT n = 8, 4-week KO n = 7, 7-week WT n = 11, 7-week KO n = 13). (L) Latency to fall from an accelerating rotarod measured every week from 4 weeks to 8 weeks of age of WT (n = 10) and *Mecp2* KO (n = 7) mice (*p<0.05; **p<0.01; two-way repeated measures ANOVA with Bonferroni *post hoc* test). All graphs represent mean±s.e.m.

We first characterized the consequences of MeCP2 deficiency on overall energy homeostasis in mice on standard chow diet. We used male mice on a mixed genetic background (129, C57BL6, BALB/c) that were WT or hemizygous for *Mecp2* KO. At 4 weeks of age, there were no differences in body weight (Figure 1B) or epididymal white adipose tissue (WAT) mass between WT and *Mecp2* KO mice on chow (Figure 1E). However, by 7 weeks of age, although KO mice still had body weights similar to WT controls (Figure 1A and 1C), KO mice had heavier epididymal fat pads compared to WT (Figure 1D and 1F), suggesting greater adiposity. Whole body composition analysis at 7 weeks of age by quantitative NMR confirmed that KO mice had greater fat mass than WT mice, which was accompanied by decreased lean mass (Figure 1G and 1H), to result in similar body weights. To investigate this overall imbalance, we monitored *Mecp2* KO mice and WT controls with indirect calorimetry and measured food intake and physical activity simultaneously. *Mecp2* KO and WT mice had similar levels of energy intake and overall energy expenditure (EE) rates on a standard chow diet (Table 1), consistent with the finding of similar body weights for these mice (Figure 1A and 1C). Rates of oxygen consumption, typically the main index of EE rate, were also similar between the genotypes (VO_2_, Table 1). *Mecp2* KO mice had increased respiratory exchange ratio (RER) compared with WT controls (Table 1), suggesting a whole-body shift in fuel utilization either away from fat oxidation, or alternatively further toward carbohydrate utilization on the chow diet. In the context of the increased adiposity of the KO mice, the former interpretation is most likely. Notably, RER was increased in KO versus WT, but VO_2_ was similar between the genotypes, despite a significantly lower level of physical activity in KO mice (Table 1), indicating that the increased RER in the KO could not be due to oxygen debt incurred from increased activity.

**Table 1:** Energy balance in chow-fed WT and *Mecp2* KO mice.

	Energy intake (kcal)	EE (kcal/kg/hr)	VO_2_ (ml/kg/hr)	Activity (beam breaks)	RER
Chow WT	11.6±0.6	15.4±0.5	3040.7±96.2	35598.4±2408.6	1.003±0.007
Chow KO	11.5±0.4	15.2±0.4	2983.9±81.0	25758.0±1598.6 *	1.039±0.007 *

Values shown are mean±s.e.m.

EE, energy expenditure; RER, respiratory exchange ratio

*p<0.01.

Next, we measured metabolic biomarkers in serum in a separate cohort. Leptin level was elevated in KO mice versus WT (Figure 1I), as was leptin mRNA in epididymal WAT (Figure 1J). Although insulin levels did not show significant differences, we observed a trend at 7 weeks of age for increased serum insulin in *Mecp2* KO mice compared to WT (Figure 1I). Serum cholesterol was higher in 7 week-old KO mice compared to 4 week-old KO mice, and serum levels of triglycerides were increased at 7 weeks of age in KO mice compared to the same age WT littermates (Figure 1K). In a third cohort used for rotarod tests, no differences in body weight were observed between the genotypes during weeks 4–8 of age (data not shown). During this time, *Mecp2* KO mice averaged significantly shorter times on an accelerating rotarod, confirming that MeCP2 deficiency caused deficiencies in balance and motor coordination (Figure 1L).

### Increased survival of triheptanoin-fed *Mecp2* KO mice

**Figure 2:**
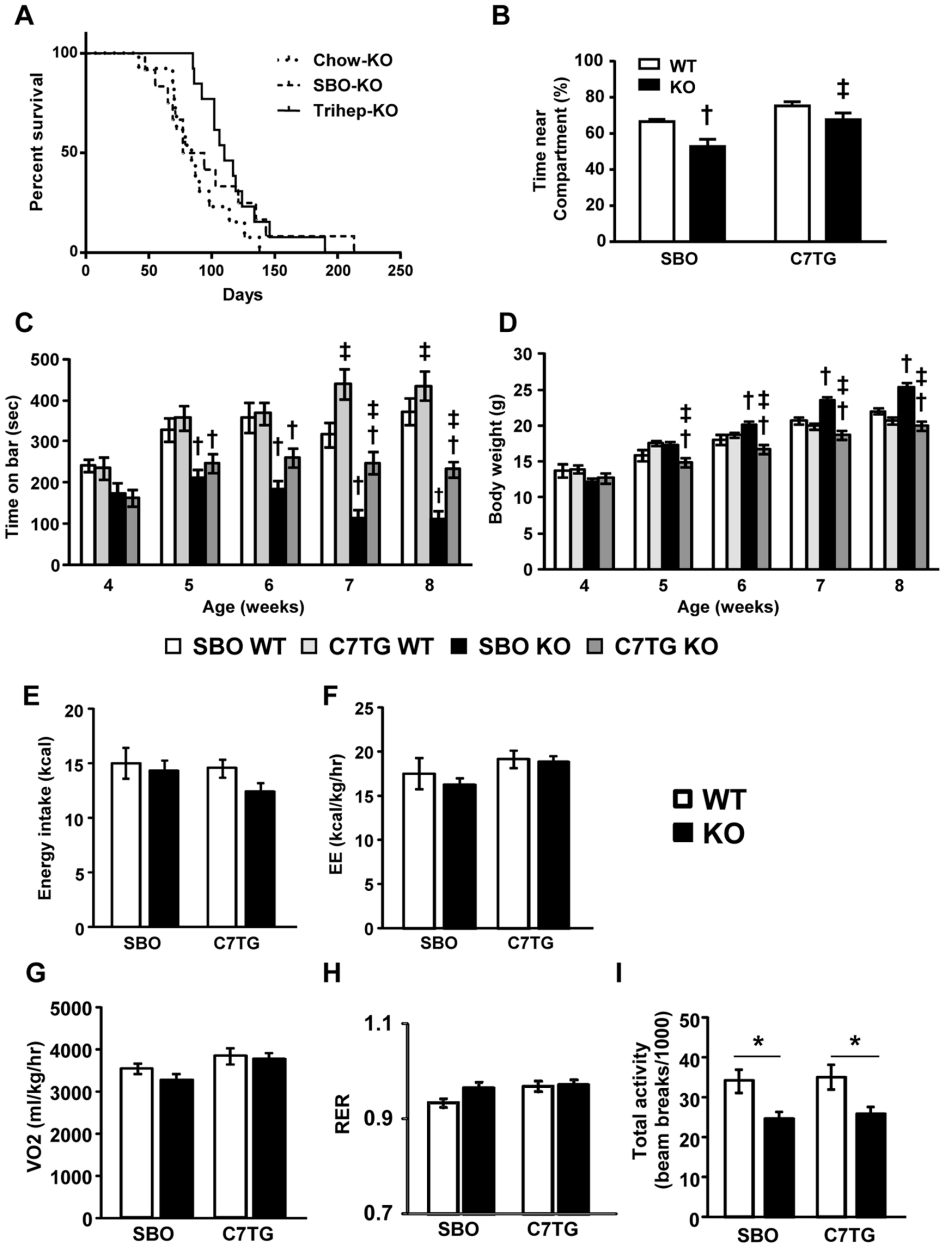
Triheptanoin-fed *Mecp2* KO mice have increased longevity, improved sociability and rotarod performance, and altered whole-body metabolism, compared to SBO-fed *Mecp2* KO mice. (A) Kaplan-Meier survival curves of triheptanoin-fed *Mecp2* KO (n = 14) compared to SBO-fed KO (n = 12) and chow-fed KO (n = 13) (Gehan-Breslow-Wilcoxon tests for curve comparisons: chow KO vs. SBO KO, p = 0.8074, NS; C7TG KO vs. SBO KO, p = 0.1502, NS; C7TG KO vs. chow KO, p = 0.0087). (B) Test for social interaction without direct physical contact; time spent by test mouse in the half of a home cage-like environment nearest to a novel mouse (two-way ANOVA with Tukey *post hoc* test; dagger symbol, differs from matching WT control; double dagger, differs from matching SBO control; p<0.05; SBO WT n = 11, C7TG WT n = 11, SBO KO n = 10, C7TG KO n = 10). (C) Time to fall from an accelerating rotarod, from 4 weeks to 8 weeks of age (two-way ANOVA with Tukey *post hoc* test; dagger symbol, differs from matching WT control; double dagger, differs from matching SBO control; p<0.05; SBO WT n = 19, C7TG WT n = 19, SBO KO n = 20, C7TG KO n = 19). (D) Body weights of mice in the rotarod study, fed either SBO or triheptanoin diet from 4 weeks to 8 weeks of age (two-way ANOVA with Tukey *post hoc* test; dagger symbol, differs from matching WT control; double dagger, differs from matching SBO control; p<0.05). (E-I): Energy intake (E), Energy expenditure (EE) (F), VO2 (G), RER (H), and physical activity (I) of mice receiving a SBO or triheptanoin diet at 8 weeks of age (two-way ANOVA and Tukey *post hoc* test, *p<0.05; n = 7-8 per group). Graphs (B-I) represent data as mean±s.e.m.

*Mecp2* KO mice and WT controls were switched from chow diet to either a triheptanoin-supplemented diet or isocaloric soybean oil (SBO) control diet at 4 weeks of age. At 7 weeks old, after 3 weeks of diet intervention, survival curves of the triheptanoin-KO and SBO-KO groups began to diverge. First deaths in the chow-KO and SBO-KO groups were at 42 and 47 days respectively, whereas the first triheptanoin-KO mouse died at day 85. (Figure 2A). Median lifespan of triheptanoin-KO mice was 110 days, versus 85.5 days for SBO-KO and 84 days for chow-KO. Until these median time points, rates of deaths were the same across groups. After these median time points, remaining SBO-KO mice appeared to die at a slower rate. The triheptanoin-KO survival curve was significantly different from chow-KO (χ^2^ = 6.883, p = 0.0087, hazard ratio = 2.566), and tended to differ from the curve for SBO-KO (χ^2^  = 2.07, p = 0.1502, hazard ratio = 1.221). Curves for chow-KO and SBO-KO did not differ (χ^2^ = 0.05944, p = 0.8074, hazard ratio = 1.459). All WT mice survived through the end of the tests. Overall, anaplerotic therapy with dietary triheptanoin significantly increased lifespan of *Mecp2* KO mice.

### Improved social interaction, motor coordination, and energy balance *Mecp2* KO mice

Although triheptanoin increased longevity in *Mecp2* KO mice, it was important to determine whether triheptanoin diet improved RTT-like symptoms such as impaired social interaction or motor coordination. We evaluated sociability in *Mecp2* KO mice using a test not involving physical contact between the test mouse and a novel WT mouse[32]. After 4 weeks of diet treatments, KO mice that were fed SBO spent significantly less time in the cage area closest to the novel mouse, than WT mice on SBO. In contrast, KO and WT mice that were fed C7TG spent the same amount of time near the novel mouse (Figure 2B). KO mice on triheptanoin diet spent significantly more time near the novel mouse than KO mice on control SBO diet (Figure 2B). To examine motor competence, we measured latency to fall off an accelerating rotarod on a weekly basis in mice during 4 to 8 weeks of age while on test and control diets (Figure 2C). The triheptanoin-fed *Mecp2* KO mice maintained their baseline motor abilities, while the SBO-KO group's performance declined over time. We did note that while SBO was not obesogenic in the WT mice during the 4-week treatment, *Mecp2* KO mice gained significant body weight on SBO (Figure 2D). We performed a correlation analysis, and found no relationship between body weight and time on rotarod (p = 0.5636), indicating that the decline in rotarod performance in the SBO-KO group was not due to weight gain.

Triheptanoin diet, despite having the same amount of kcals from fat as SBO, did not cause excessive weight gain in mice of either genotype. To determine whether the increase in body weight in the SBO-KO group versus triheptanoin-KO was due to increased energy intake or decreased EE, mice were monitored by indirect calorimetry with simultaneous measurements of food intake and physical activity. Daily energy intakes were not significantly different across genotype or diet groups (Figure 2E). Rates of EE were affected significantly by diet (main diet effect, p = 0.011); although post-hoc tests did not reveal significant group differences between genotypes, the SBO-fed groups had tended to have lower EE than triheptanoin-fed groups; thus, triheptanoin diet enhanced EE in both genotypes versus the SBO (Figure 2F). This may explain why Mecp2 KO mice maintained WT-like body weights over time on triheptanoin diet versus SBO. Analyzing the components of EE revealed that VO_2_ was enhanced in triheptanoin-fed mice versus SBO-fed mice of both genotypes (Figure 2G, main diet effect, p = 0.014), whereas RER showed no significant differences across genotype or diet (Figure 2H). As with KO mice fed chow (Table 1), *Mecp2* KO mice had low levels of physical activity which was not affected by either SBO or triheptanoin (Figure 2I). Thus, although triheptanoin improved rotarod performance in *Mecp2* KO mice, it did not normalize levels of spontaneous physical activity within the calorimeter cage environment. However, despite low physical activity in KO mice, the triheptanoin diet resulted in increased VO_2_ and EE versus SBO, indicating that the increase in baseline metabolic rate incurred with triheptanoin could not be due to increased activity, supporting the conclusion that triheptanoin diet supports a general enhancement of basal oxidative metabolism.

### Decreased serum leptin and insulin levels in triheptanoin-fed *Mecp2* KO mice

**Figure 3:**
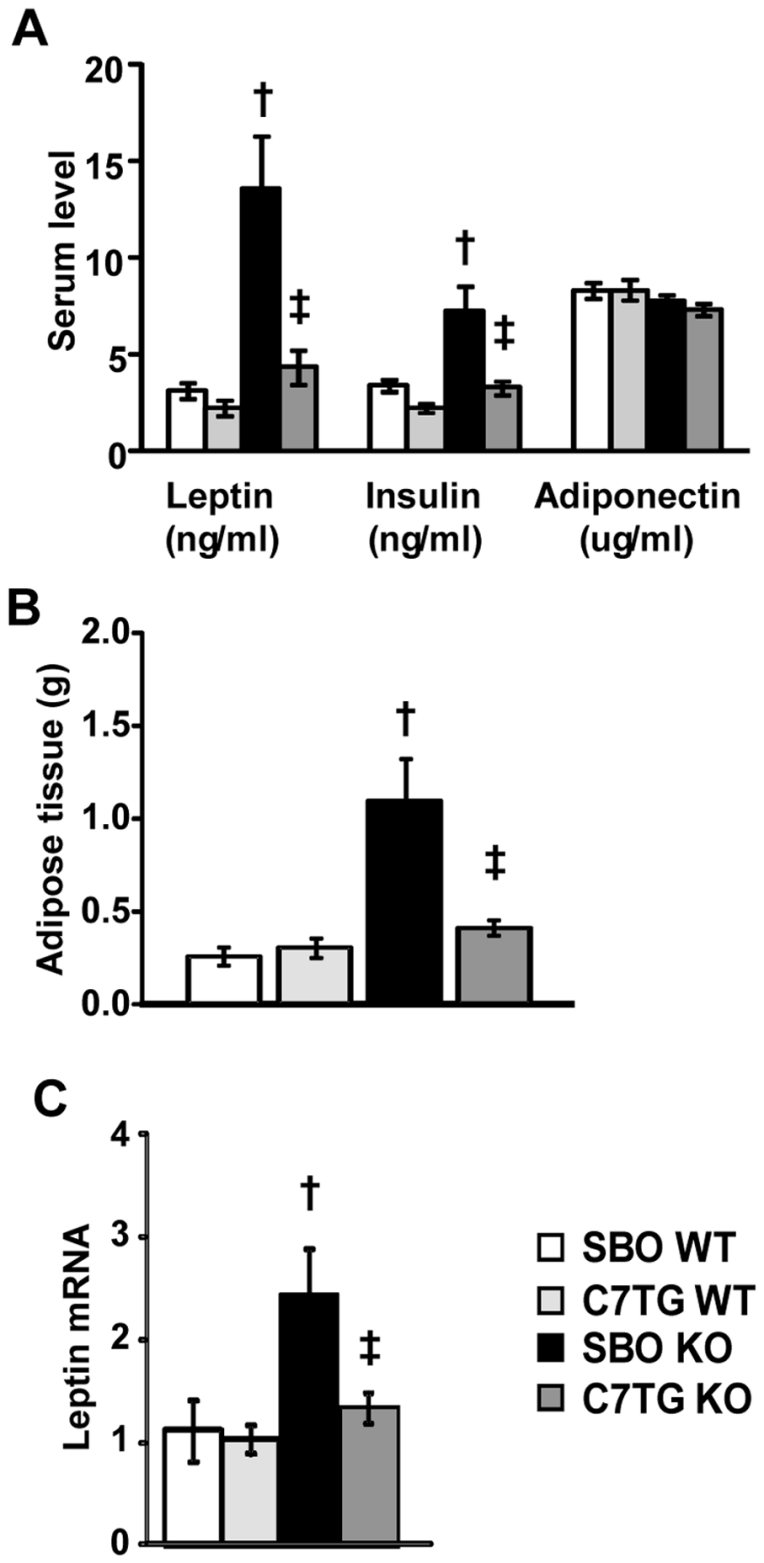
Triheptanoin lowers leptin and insulin levels in *Mecp2* KO mice. (A) Serum levels of leptin, insulin, and adiponectin measured by Multiplex assay at 8 weeks of age in *Mecp2* KO mice and WT littermates (SBO WT n = 14, C7TG WT n = 12, SBO KO n = 15, C7TG n = 14). (B) Weight of WAT in SBO- or triheptanoin-fed WT and KO mice (SBO WT n = 6, C7TG WT n = 4, SBO KO n = 6, C7TG KO n = 5). (C) Expression levels of *leptin* mRNA from WAT, data for each group are fold-changes relative to SBO WT control with actin as a reference gene (SBO WT n = 9, C7TG WT n = 11, SBO KO n = 10, C7TG KO n = 8). For all panels A-C, two-way ANOVA with Tukey *post hoc* test; dagger symbol, differs from matching WT control; double dagger, differs from matching SBO control; p<0.05. All graphs represent data as mean±s.e.m.

In humans, RTT is associated with metabolic abnormalities that include increased leptin and decreased adiponectin[19], [20], and an *Mecp2* KO mouse model of RTT has been shown recently to have a metabolic syndrome that includes hyperinsulinemia[33]. Chow-fed *Mecp2* KO had increased leptin, and a tendency for increased insulin, resembling findings in RTT patients (Figure 1I). *Mecp2* KO mice on SBO diet, to an even greater degree than chow-fed KO, had hyperleptinemia and significant hyperinsulinemia (Figure 3A). Triheptanoin diet resulted in significantly lower levels of leptin and insulin in *Mecp2* KO, despite having the same percent of kcals from fat as SBO diet, resulting in normal levels of leptin and insulin similar to those seen in WT (Figure 3A). Triheptanoin-treated KO mice had decreased epididymal WAT mass (Figure 3B) and lower leptin mRNA in epididymal WAT (Figure 3C) compared to SBO-KO mice, and similar to WAT and leptin mRNA levels of WT. Thus, despite the higher level of dietary fat, anaplerotic therapy with triheptanoin did not increase adiposity or produce hyperleptinemia or hyperinsulinemia.

### Increased glucose tolerance and insulin sensitivity in triheptanoin-fed *Mecp2* KO mice

**Figure 4:**
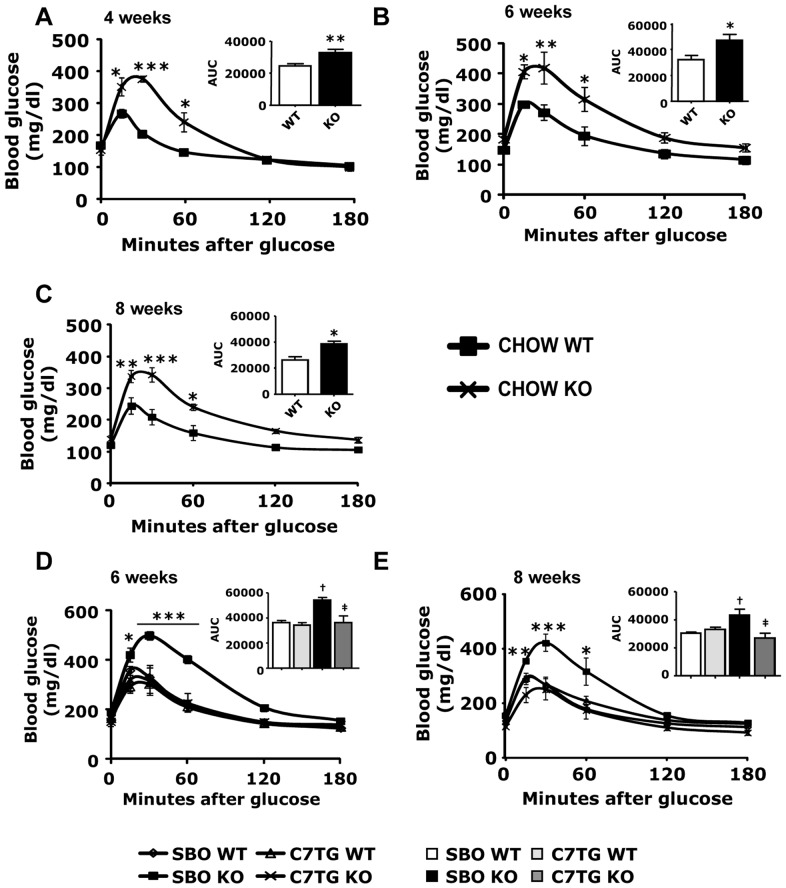
Triheptanoin diet improves glucose sensitivity in *Mecp2* KO mice. (A–C): Blood glucose levels after intraperitoneal glucose injection (1 mg/kg) at 4 weeks (A), 6 weeks (B), and 8 weeks of age (C) in chow-fed WT or *Mecp2* KO mice (WT vs. KO *p<0.05, **p<0.01, ***p<0.001; two-way ANOVA with Bonferroni *post hoc* test; 4wk CHOW WT n = 6, 4 wk CHOW KO n = 8). (D, E): Blood glucose levels after intraperitoneal glucose injection (1 mg/kg) at 6 weeks (D) and 8 weeks (E) of age after two (D) or four (E) weeks of SBO or triheptanoin diet in WT or *Mecp2* KO mice (SBO-KO vs. other groups *p<0.05, **p<0.01, ***p<0.001; two-way ANOVA with Bonferroni *post hoc* test; n = 4 per group). Inserts represent areas under the curves (AUC). For AUC inserts of panel D and E, dagger symbols denote differences from matching WT control, double daggers denote differences form matching SBO control. All graphs represent data as mean±s.e.m.

The adiposity and serum adipokine data suggested that *Mecp2* KO mice could exhibit an adiposity-related, type-II diabetic profile. We first conducted intraperitoneal (IP) glucose tolerance tests (GTT) on chow-fed WT and *Mecp2* KO mice at 4, 6, and 8 weeks of age. Chow-fed KO mice exhibited decreased glucose tolerance at all ages (Figure 4 A–C). We note that within this context of poor glucose tolerance, the likelihood that the increased RER of chow-fed *Mecp2* KO mice (Table 1) indicated increased carbohydrate oxidation does not seem tenable; thus that increase in RER probably indicated lower fat oxidation, as suggested by the fatty phenotype of *Mecp2* KO. The data overall support the hypothesis and indicate that *Mecp2* KO mice have an adiposity-related diabetes.

The adiposity and serum cytokine data also indicated that anaplerotic therapy with dietary triheptanoin would prevent the type-II diabetic profile seen in *Mecp2* KO. Therefore, IP GTT was also conducted in SBO- or triheptanoin-fed WT and *Mecp2* KO mice at 6 and 8 weeks of age, to determine whether triheptanoin could ameliorate impaired glucose tolerance. SBO-fed KO mice, like chow-fed KO, had significantly elevated blood glucose for over an hour post-injection. Triheptanoin-fed KO mice exhibited noticeably lower blood glucose, similar to WT groups on both triheptanoin and SBO diets, for the duration of the test (Figure 4D and 4E).

**Figure 5:**
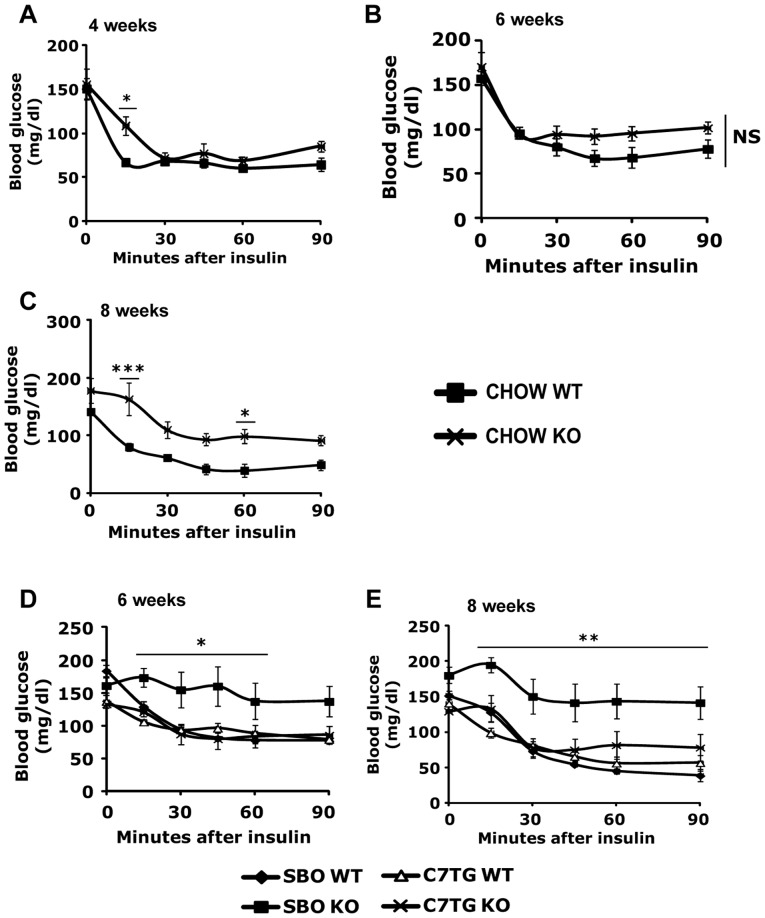
Triheptanoin diet improves insulin sensitivity in *Mecp2* KO mice. (A–C): Blood glucose levels after intraperitoneal insulin injection (1 unit/kg) at 4 weeks (A), 6 weeks (B), and 8 weeks of age (C) in chow-fed WT or *Mecp2* KO mice (WT vs. KO *p<0.05, **p<0.01, ***p<0.001; two-way ANOVA with Bonferroni *post hoc* test; 4 wk CHOW WT n = 6, 4 wk CHOW KO n = 8. (D, E): Blood glucose levels after intraperitoneal insulin injection (1 unit/kg) at 6 weeks (D) and 8 weeks (E) of age after two (D) or four (E) weeks of SBO or triheptanoin diet in WT or *Mecp2* KO mice (SBO-KO vs. other groups *p<0.05, **p<0.01, ***p<0.001; two-way ANOVA with Bonferroni *post hoc* test; n = 4 per group). All graphs represent mean±s.e.m.

We also assessed insulin sensitivity with IP insulin tolerance tests (ITT) on WT and *Mecp2* KO mice exposed to the different diets. Blood glucose levels were measured after IP insulin injection. Both chow-fed (Figure 5A–C) and SBO-fed KO mice (Figure 5D and 5E) exhibited insulin resistance compared with the WT groups, as indicated by higher blood glucose concentrations after the insulin bolus. Triheptanoin diet resulted in normal insulin sensitivity in *Mecp2* KO mice (Figure 5D and 5E). In all, triheptanoin diet appeared to prevent a type-II diabetes profile in *Mecp2* KO mice.

**Figure 6:**
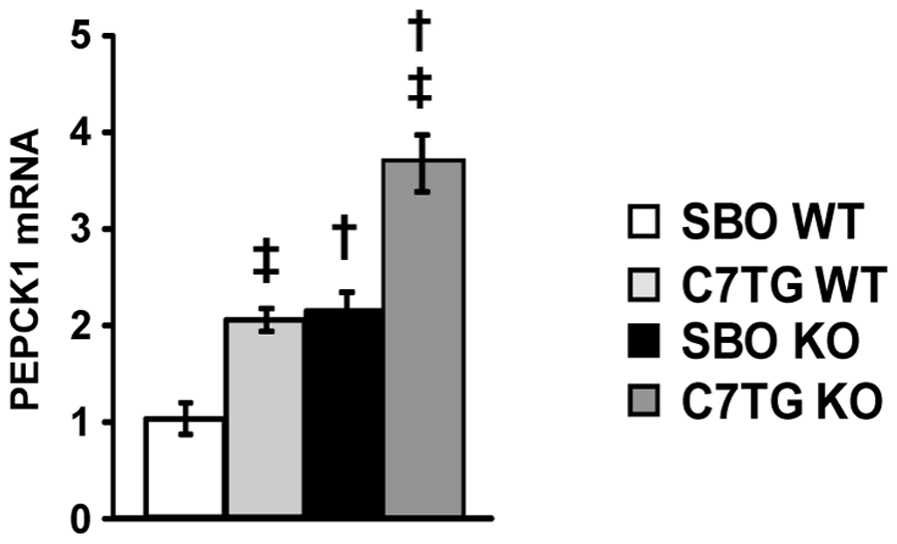
*Mecp2* KO mice have increased gene expression for PEPCK in liver; separate effect of triheptanoin. Expression levels of *pepck1* mRNA from liver, data for each group are fold-changes relative to SBO WT control with actin as a reference gene (two-way ANOVA with Tukey *post hoc* test; dagger symbols denote differences from matching WT controls, double daggers denote differences from matching SBO controls, p<0.05; SBO WT n = 4, C7TG WT n = 5, SBO KO n = 5, C7TG n = 5).

Liver phosphoenolpyruvate carboxykinase 1 (PEPCK) is well-known as the rate-limiting enzyme for gluconeogenesis, an important function of liver during starvation, but also a contributor to hyperglycemia in type-II diabetes[34]. Because *Mecp2* KO mice on either chow or SBO control diets displayed hyperglycemia and insulin resistance, we hypothesized that KO mice on a control diet would have increased expression for liver PEPCK compared with the WT mice. Further, because triheptanoin diet prevented the hyperglycemia and insulin resistance in KO mice, we hypothesized that part of the mechanism by which triheptanoin prevents type-II diabetes in KO would be decreased or normalized liver PEPCK expression. We measured levels of mRNA for PEPCK (cytosolic) in livers, from *Mecp2* KO and WT mice on triheptanoin diet or SBO control diet. There were significant effects of genotype, and of diet, but with no significant interaction effect. As hypothesized, *Mecp2* KO mice on SBO control diet had significantly higher mRNA expression for liver PEPCK than WT (Figure 6). Surprisingly, KO mice on the triheptanoin diet had higher mRNA for PEPCK than WT controls on triheptanoin, i.e. triheptanoin did not normalize PEPCK expression in *Mecp2* KO to WT baseline. Furthermore, in WT mice, triheptanoin diet resulted in increased PEPCK expression as well versus SBO control. Although PEPCK is important for gluconeogenesis, it is also present in other tissues that do not ordinarily make glucose[35]. A broader role of PEPCK in all tissues may be cataplerosis, the removal of the excess TCA cycle anions from mitochondrial matrix that could occur from anaplerosis[27]. Thus, the increased PEPCK gene expression in response to triheptanoin diet, regardless of mouse genotype, may serve to offset and balance the diet-induced anaplerosis.

### Triheptanoin diet improves mitochondrial morphology and content in skeletal muscle of *Mecp2* KO mice

**Figure 7:**
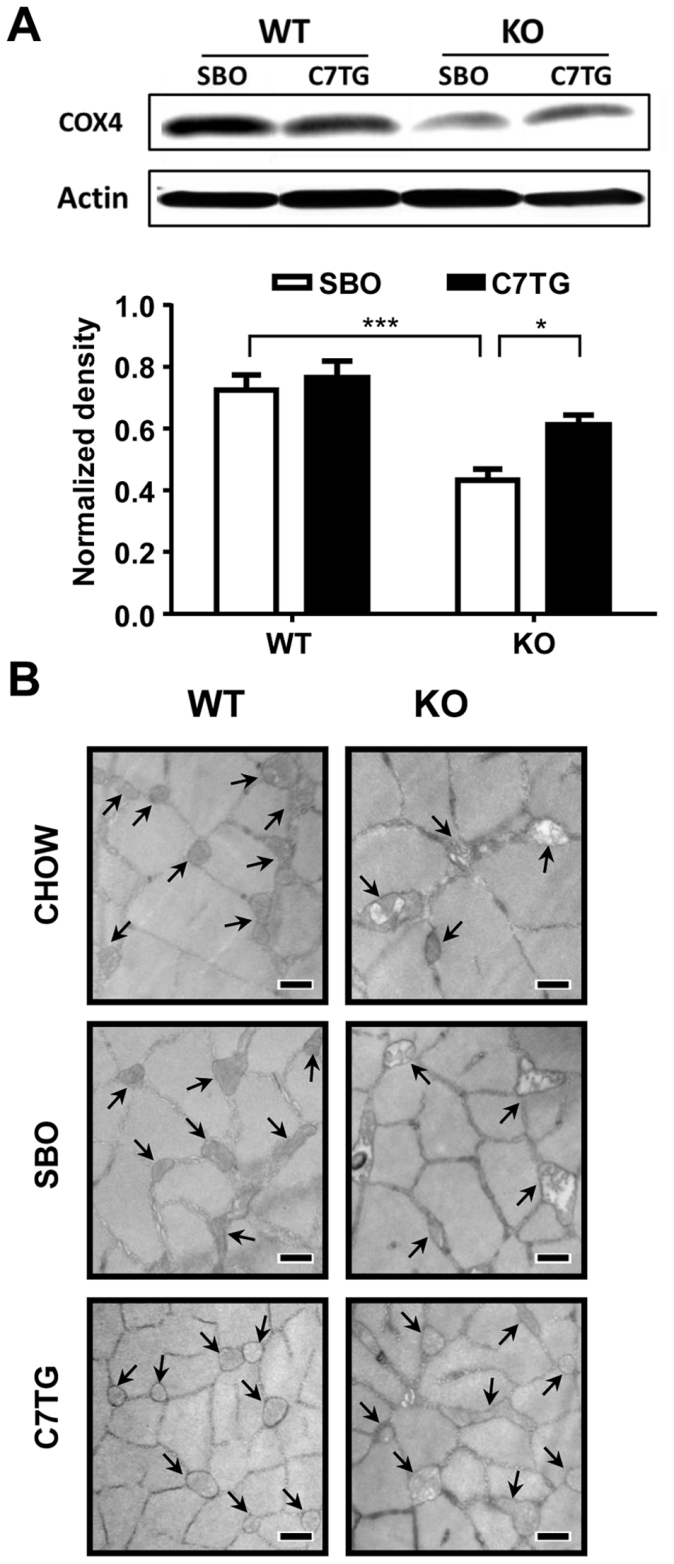
Triheptanoin improves mitochondrial morphology in skeletal muscle of *Mecp2* KO mice. (A) Levels of a mitochondrial biogenesis marker, cytochrome oxidase 4 (COX4), were measured using Western blot in skeletal muscles (gastrocnemius, plus soleus and plantaris). Quantitation of COX4 blots (n = 6 per group); two-way ANOVA with Tukey *post hoc* test, asterisks show significant group differences, *p<0.05, ***p<0.001. (B) Representative transmission electronic microscopy images of gastrocnemius muscle at 8 weeks of age (10,000 X, scale bar = 500 nm). Arrow indicates mitochondrion.

Mitochondrial dysfunction has been reported in patients with RTT and other ASDs[14]–[18], [36]–[39], and is associated with impaired motor function[40] as well as glucose and insulin resistance[41]. We hypothesized that mitochondrial abnormalities contribute to the metabolic imbalance, and may also contribute to the altered motor coordination seen in MeCP2 deficient mice. In this context, anaplerotic dietary treatment with triheptanoin would improve mitochondrial impairment, as it did for the deficits in motor coordination (Figure 2B), metabolism (Figure 3), and lifespan (Figure 2A). To index mitochondrial content in skeletal muscles (gastrocnemius, plus soleus and plantaris), we performed western blots for cytochrome c oxidase subunit 4 (COX4), a nuclear-encoded subunit of cytochrome oxidase c, the terminal enzyme of the electron transport chain on the inner mitochondrial membrane (Figure 7A). Quantitation of the WB densities showed significant effects of both genotype and diet. SBO-fed KO showed decreased COX4 compared to WT controls. Triheptanoin-fed KO displayed significantly higher level of COX4 than SBO-fed KO, and not significantly less than WT controls. Transmission electron microscopy of muscle from chow-fed *Mecp2* KO revealed that most mitochondria were enlarged and misshapen compared with mitochondria in muscle from chow-fed WT mice (Figure 7B). *Mecp2* KO mitochondria in muscle had electron-lucent central matrices and non-parallel, disorganized cristae; mitochondria in muscle from SBO-fed *Mecp2* KO appeared even more dysmorphic. In contrast, mitochondria from skeletal muscle of triheptanoin-fed *Mecp2* KO mice appeared normalized (Figure 7B), with more organized cristae and more electron-dense central matrix than seen in KO mice on the other diets. These results show that triheptanoin improves mitochondrial content and morphology, consistent with the hypothesis that anaplerotic diet therapy may promote energy production[15]–[18], [39].

### Altered metabolite profiles in triheptanoin-fed mice support the benefits of anaplerotic diet therapy for *Mecp2* KO

**Figure 8:**
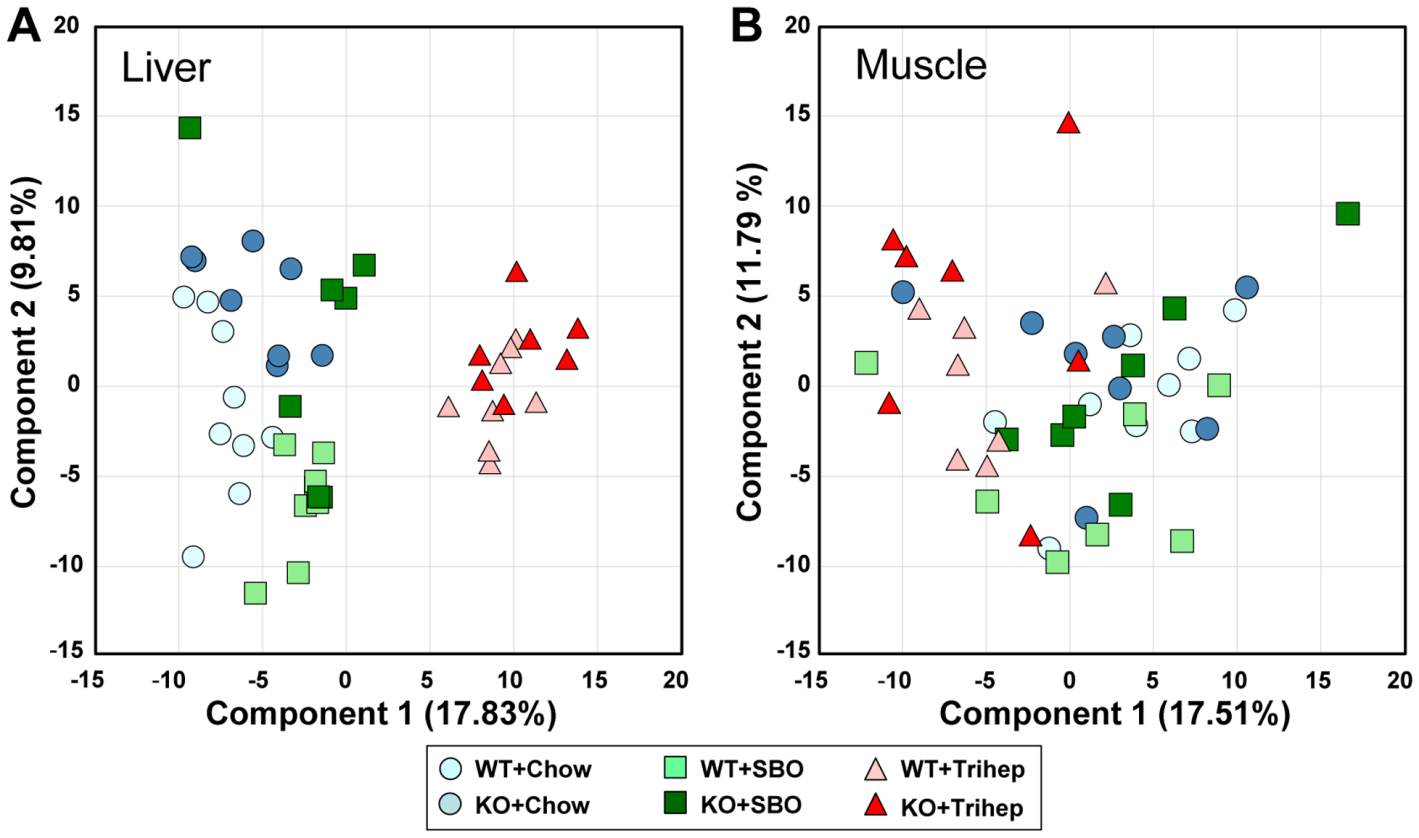
Principal component analysis shows unique metabolomic profile of mice eating triheptanoin diet. (A) Principal component analysis (PCA) score plot of liver samples from *Mecp2* KO and WT mice on triheptanoin diet and control diets. (B) PCA score plot of skeletal muscle samples from *Mecp2* KO and WT mice on triheptanoin diet and control diets.

To investigate metabolic mechanisms that may underlie the improvements in metabolism and RTT-like symptoms seen with anaplerotic triheptanoin therapy, we performed untargeted metabolomics on extracts from liver and skeletal muscle (gastrocnemius, soleus, and plantaris) of WT and *Mecp2* KO mice fed chow, triheptanoin diet, or SBO control diet. From principal component analysis (PCA) score plots of liver samples (Figure 8A) it was clear that mice on triheptanoin diet (right side of plot) had a metabolic signature that differentiated them along the dimension of principal component 1 from mice eating control diets (left side of plot). Within component 1, seven of the largest positive PCA coefficients(>0.10) were odd-carbon-length fatty acids, including heptanoate itself, or were metabolites containing odd-carbon-length fatty acids (data not shown). Within the triheptanoin diet cluster, WT and KO liver samples were not separated. The other liver samples did not show clear separation, but it appeared that analysis of WT and KO mice displayed subtle differences along the dimension of principal component 2, with the KO tending to be at the top of the plot. Within component 2, many lysolipids had large, negative PCA coefficients (<-0.10), as did glycerol and beta-hydroxybutyrate ketone body (BHBA), whereas amino acids often had large positive coefficients(>0.05) (data not shown). Muscle samples did not show clear separation by genotype or diet in the PCA score plot (Figure 8B).

**Figure 9:**
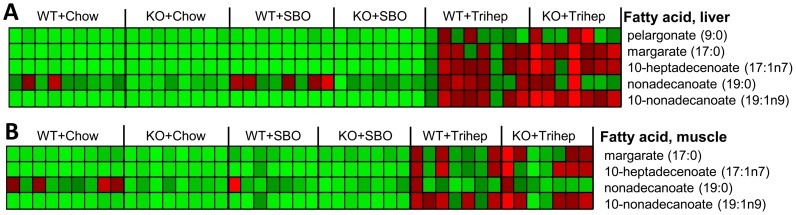
Untargeted metabolomics demonstrate production of odd-carbon-length fatty acids in mice fed triheptanoin diet. **A.** Heat map representation of relative levels of odd-carbon-length fatty acids in liver. **B.** Heat map representation of relative levels of odd-carbon-length fatty acids in skeletal muscle. SBO, soybean oil control diet. Metabolomics data were normalized to the median of the WT+chow control group, median scaled to 1, but are presented as averages in the heat maps. Row-normalized heat maps display individual samples by column and metabolites by row. Color intensity is scaled within each row so that the highest expression value corresponds to bright red and the lowest to bright green. All fatty acids in Figure 9 showed significant effects of diet by two-way ANOVA of contrasts (p <0.0001).

Heptanoate itself was only detected in tissues from mice fed triheptanoin diet. Consistent with published observations that most heptanoate from enteral administration is metabolized by first-pass through the liver[42], the raw intensity values for heptanoate in muscle were about one-third those in liver (data not shown). Levels of other odd-carbon length fatty acids were increased in the triheptanoin-fed groups in both liver (Figure 9A) and skeletal muscle (Figure 9B), compared with mice fed the control diets.

**Figure 10:**
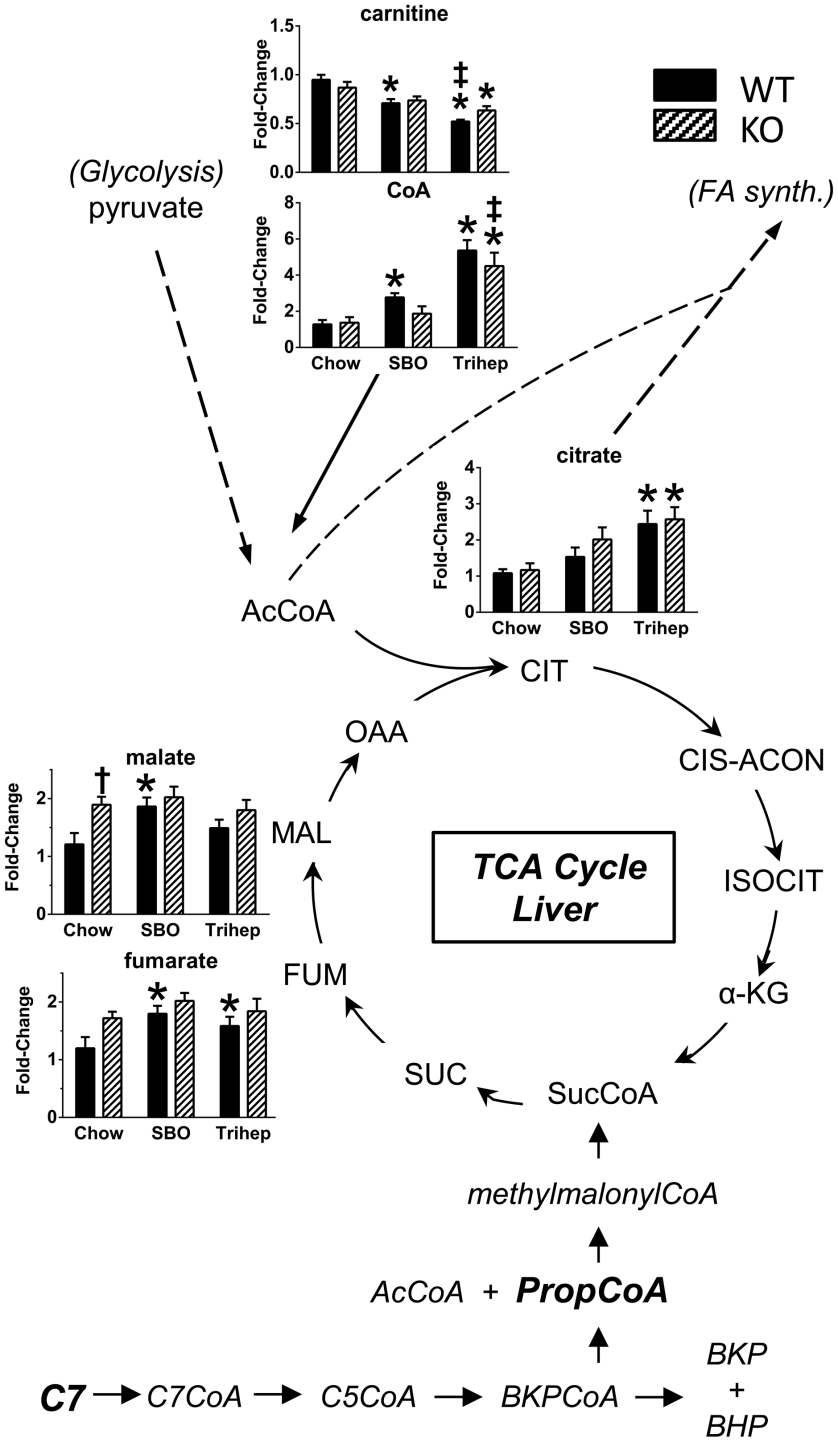
Anaplerosis of hepatic TCA cycle intermediates with triheptanoin diet. Heptanoate (C7) from triheptanoin diet (Trihep) enters the portal circulation, and is largely metabolized by liver to betaketopentanoate (BKP), then to acetyl-CoA and propionyl-CoA. Some BKP, in equilibrium with betahydroxypentanoate (BHP), is exported to the systemic circulation. Propionyl-CoA is metabolized further to provide succinyl-CoA to the TCA cycle. This is expected to increase TCA cycle flux, replenishing oxaloacetate (OAA) so that acetyl groups from acetyl-CoA fuel can enter the TCA cycle. Bar graphs of Figure 6 show metabolomics data from liver of WT (black bars) and *Mecp2* KO mice (hatched bars), on either chow, a high-soybean oil control diet (SBO), or anaplerotic triheptanoin diet. Triheptanoin diet resulted in significantly elevated levels of free CoA. Combined with increased TCA cycle flux, this permitted increased hepatic citrate in mice on triheptanoin diet. Data in bar graphs were normalized to the median of the WT+chow control group, median scaled to 1, but are presented as averages with standard errors. Symbols denote group differences by ANOVA contrast comparisons of log-transformed data (p≤0.05): asterisk, differs from matching chow control, dagger, differs from matching WT control; double dagger, differs from matching SBO control.

The metabolic fate of dietary triheptanoin[26], [29], [42] in liver is illustrated in the lower portion of Figure 10. Heptanoate (C7) from dietary triheptanoin enters the portal circulation to liver, where it is esterified to CoA and metabolized to the CoA ester of five-carbon-ketone beta-ketopentanoate (BKP, in equilibrium with beta-hydroxypentanoate, BHP), and then to acetyl-CoA and propionyl-CoA. Propionyl-CoA is metabolized by additional enzymes to provide succinyl-CoA, an intermediate of the TCA cycle. This anaplerotic effect is expected to increase TCA cycle flux and replenish oxaloacetate (OAA) so that acetyl groups can enter the TCA cycle at citrate synthase[23], [24], [26]. The bar graphs in Figure 10 show metabolomics data from livers of WT and *Mecp2* KO mice fed chow, SBO, or the anaplerotic triheptanoin diet. Chow-fed *Mecp2* KO mice had higher hepatic levels of the late-TCA cycle intermediates malate and fumarate compared to WT controls (Figure 10). However, levels of citrate were not increased in chow-fed KO, so the elevated malate and fumarate may reflect low levels of OAA replenishment. Triheptanoin diet did result in elevated hepatic levels of citrate (Figure 10), in both WT and KO, suggesting an increased TCA cycle flux as outlined above. Triheptanoin treatment was also characterized by increased hepatic free CoA (Figure 10), which would be available to make acetyl-CoA, and accommodate entry of acetyl groups into the TCA cycle and the increased citrate. The ratio of acyl-CoAs to free CoA is buffered by carnitine[43], [44]; in livers of triheptanoin-fed mice, the levels of free carnitine were decreased, reciprocal to the diet-dependent pattern observed for free CoA.

**Figure 11:**
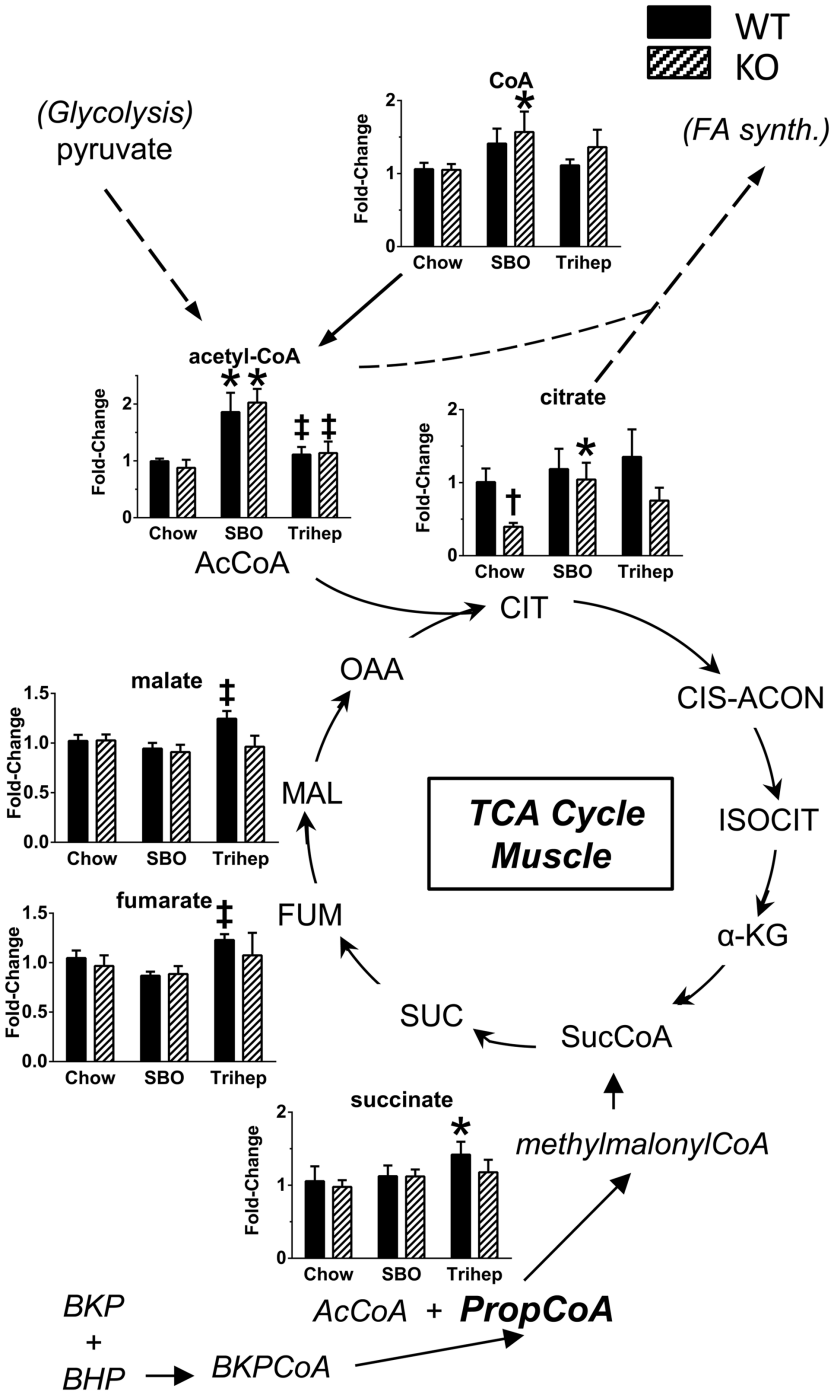
Anaplerosis of skeletal muscle TCA cycle intermediates with triheptanoin diet. BKP and BHP from liver metabolism of heptanoate enter muscle from the systemic circulation, and are metabolized to anaplerotic propionyl-CoA, plus acetyl-CoA. Bar graphs in Figure 6B show metabolomics data from gastrocnemius (plus soleus and plantaris) of WT and *Mecp2* KO mice on chow, high-soybean oil control diet (SBO), or triheptanoin diet (Trihep). Triheptanoin diet resulted in increased steady-state levels of late-TCA cycle intermediates succinate, fumarate, and malate versus SBO controls, evident in the WT mice with normal baseline TCA cycle function, indicating increased late-TCA cycle flux. SBO-fed mice of both genotypes showed elevated levels of muscle acetyl-CoA versus chow, whereas mice fed triheptanoin diet (isocaloric and fat-normalized with SBO) did not, suggesting more efficient entry of acetate carbons into the TCA cycle with anaplerotic diet. Data in bar graphs were normalized to the median of the WT+chow control group, median scaled to 1, but are presented as averages with standard errors. Symbols denote group differences by ANOVA contrast comparisons of log-transformed data (p≤0.05): asterisk, differs from matching chow control, dagger, differs from matching WT control; double dagger, differs from matching SBO control.

Some hepatic BKP from triheptanoin metabolism is exported to the systemic circulation, from which BKP is available to other tissues such as skeletal muscle to provide energy[26], [29], [42]. A minor portion of dietary heptanoate escapes liver metabolism, and could also be available to other tissues and converted locally to BKP. BKP is esterified to CoA and metabolized, as in liver, to propionyl-CoA which provides succinyl-CoA to the TCA cycle. Figure 11 shows metabolomics data of TCA cycle intermediates and associated molecules detected in skeletal muscle extracts from WT and *Mecp2* KO mice fed chow, SBO, or anaplerotic triheptanoin diet. In muscle, triheptanoin diet resulted in elevated levels of the late-TCA cycle intermediates succinate, fumarate, and malate (Figure 11), evident in the WT mice. It is important to note that WT mice have normal mitochondria and TCA cycle flux; hence it is possible that anaplerosis from BKP increased the levels of late-TCA cycle intermediates in WT muscle. An unchanged level of late-TCA cycle anions in the *Mecp2* KO mice does not rule out increased flux through that part of the cycle, and indeed may be the result of increased flux toward replenished OAA. In muscle, *Mecp2* KO mice had lower levels of citrate than WT controls, suggesting low TCA cycle flux and impaired replenishment of OAA for acetyl-CoA entry into the cycle (Figure 11). Compared to chow diet, triheptanoin diet tended to augment citrate levels in both genotypes. In muscle, the SBO diet, rich in long-chain even-carbon fatty acids, produced elevated levels of two-carbon acetyl-CoA and CoA (Figure 11), which may have allowed some citrate formation in SBO-fed KO versus chow control. Importantly, however, the SBO diet did not improve the general phenotype of *Mecp2* KO mice, so citrate level *per se* may not be the key to improvements seen with triheptanoin diet.

**Figure 12:**
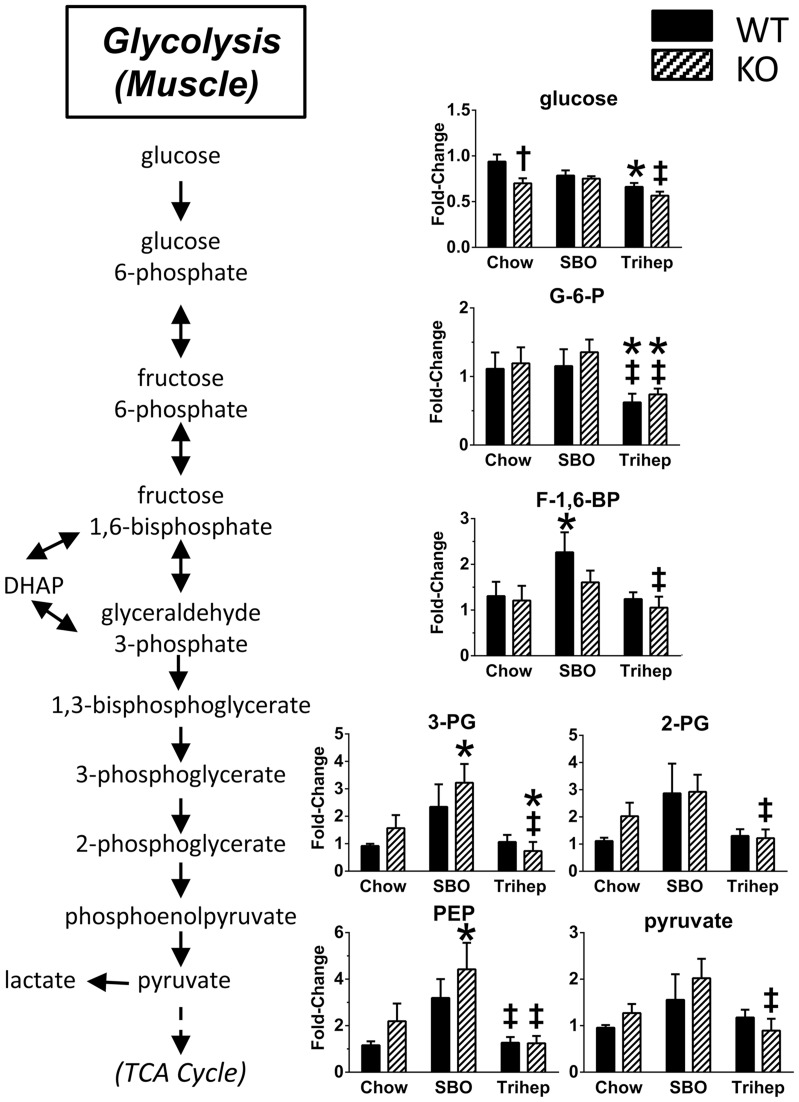
Anaplerotic triheptanoin diet permits efficient glycolysis in skeletal muscle. Mice fed the high-soybean oil control diet (SBO) mice had elevated muscle levels of late-glycolytic intermediates (fructose-1,6-bisphosphate, 3-phosphoglycerate, 2-phosphoglycerate, phosphoenolpyruvate, and pyruvate), whereas triheptanoin-fed mice (Trihep) did not. This pattern was the same as seen for acetyl-CoA in muscle (Figure 6), suggesting a back-up of glycolysis due to inefficient entry of acetate carbons in the SBO-fed mice. The early-glycolytic metabolites (glucose and G-6-P) were decreased in triheptanoin-fed mice, suggesting enhanced glucose uptake and metabolism in triheptanoin-fed mice. Data in bar graphs were normalized to the median of the WT+chow control group, median scaled to 1, but are presented as averages with standard errors. Symbols denote group differences by ANOVA contrast comparisons of log-transformed data (p≤0.05): asterisk, differs from matching chow control, dagger, differs from matching WT control; double dagger, differs from matching SBO control.

Acetyl-CoA can come from pyruvate, product of glycolysis. In liver, there were no remarkable effects of genotype or diet on levels of glycolytic intermediates (or gluconeogenic intermediates, data not shown). However, skeletal muscle showed diet-dependent changes in levels of glycolytic intermediates. Generally, the levels of late-glycolytic intermediates (fructose-1,6-bisphosphate, 3-phosphoglycerate, 2-phosphoglycerate, phosphoenolpyruvate, and pyruvate) were increased in the SBO-fed mice versus chow controls (Figure 12), similar to the pattern for muscle acetyl-CoA (Figure 11). Muscle from triheptanoin-fed mice of both genotypes had levels of late-glycolytic intermediates that were significantly lower than seen in SBO controls, and similar to levels in chow-fed mice (Figure 12). The overall pattern suggests that with the SBO diet, there may be a build-up of late-glycolytic intermediates due to sub-optimal entry of acetyl-CoA into the TCA cycle. Increased levels of late glycolytic intermediates may not be seen in the chow control groups compared with SBO because there is not as much acetyl-CoA available from even-carbon-length long-chain fatty acids as in SBO. Levels of late-glycolytic intermediates after triheptanoin diet were normal, similar to those detected in chow-fed groups. Interestingly, triheptanoin diet resulted in lower levels of the early-glycolytic intermediate G-6-P than seen after either SBO, or chow control diet (Figure 12), and a similar pattern was seen for glucose. Triheptanoin diet may, by permitting acetyl entry into the TCA cycle, provide enough ATP to normalize or enhance glycolysis; glycolysis requires investment of 2 ATP per molecule of glucose, to gain 4 ATP by the end of the pathway. This may allow glucose uptake and glycolysis in muscle to proceed normally or perhaps with slightly higher efficiency.

**Figure 13:**
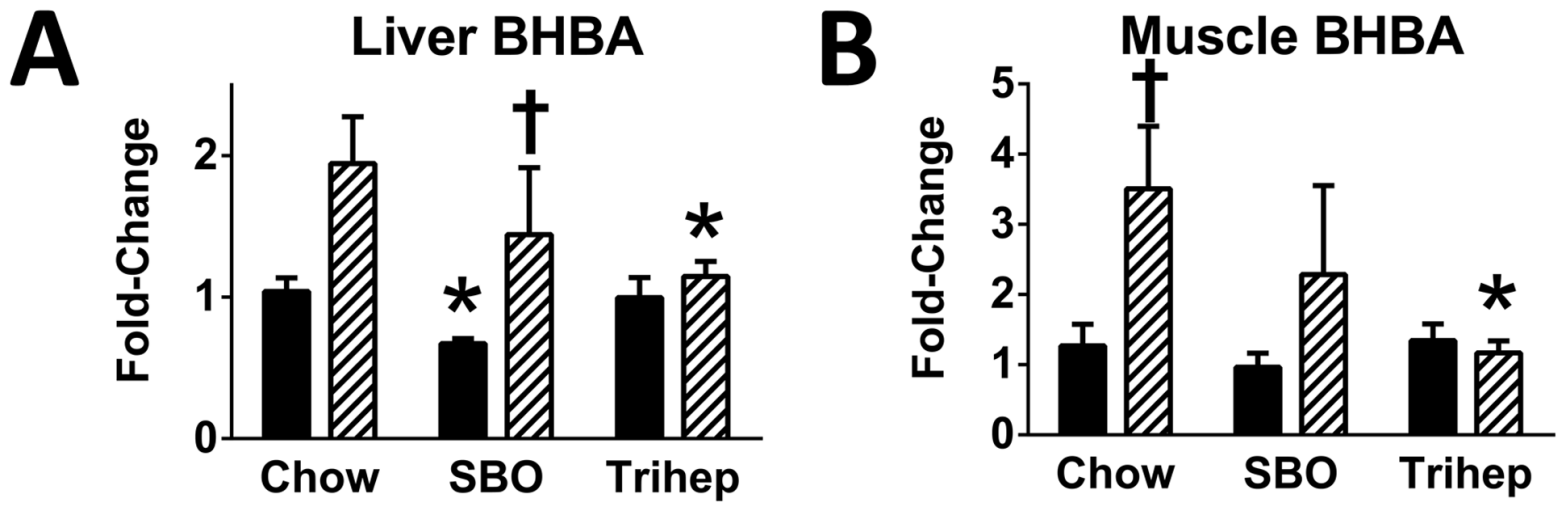
Anaplerotic triheptanoin diet normalizes steady-state betahydroxybutyrate (BHBA) in liver and muscle of *Mecp2* KO mice. *Mecp2* KO mice fed either chow or high-soybean oil control diet (SBO) had elevated levels of BHBA versus WT controls in both (A) liver and (B) skeletal muscle. *Mecp2* KO mice fed triheptanoin diet (Trihep) did not show elevated levels of this ketone body. Data in bar graphs were normalized to the median of the WT+chow control group, median scaled to 1, but are presented as averages with standard errors. Symbols denote group differences by ANOVA contrast comparisons of log-transformed data (p≤0.05): asterisk, differs from matching chow control, dagger, differs from matching WT control; double dagger, differs from matching SBO control.

A defining metabolic feature of *Mecp2* KO mice on the control diets was increased level of BHBA ketone, in both liver (Figure 13A) and skeletal muscle (Figure 13B). Elevated ketone levels are often interpreted as increased fatty acid beta-oxidation rate, in excess of the ability of the TCA cycle to accept the acetyl-CoA for immediate oxidation. However, the fatty phenotype of *Mecp2* KO mice on chow or SBO does not indicate a state of increased fatty acid oxidation (see below). Even under physiological conditions, ketones are produced at moderate levels[45]. Ordinarily, these ketones can be utilized by tissues, as they enter the TCA cycle as acetyl-CoA. However, if the processing capacity of the TCA cycle is low due to decreased levels of catalytic intermediates, acetyl-CoA may instead be directed to biosynthesis of ketone bodies[46]. Normal hepatic metabolism of heptanoate actually produces both the 5-carbon (BKP and BHP) and 4-carbon ketone bodies (BHBA). Despite this, the anaplerotic triheptanoin diet normalized BHBA levels in MeCP2 deficient tissues to levels seen in WT (Figure 13), suggesting that ketones could be utilized for fuel, again supporting the hypothesis that triheptanoin restores normal metabolic fluxes in *Mecp2* KO mice.

**Figure 14:**
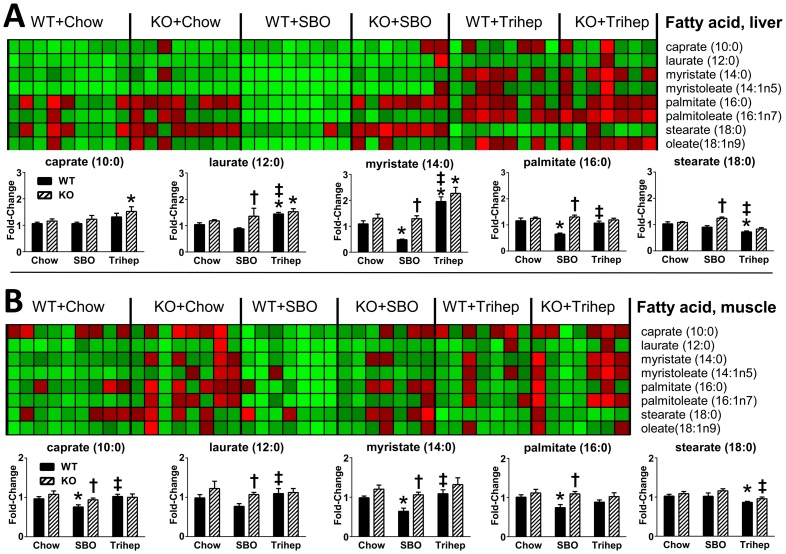
Anaplerotic triheptanoin diet enhances fatty acid oxidation in liver and skeletal muscle. **A. Liver.** Mice fed triheptanoin diet (Trihep) showed elevated levels of liver C14:0, C12:0, and C10:0 versus control diets, suggesting beta-oxidation. Hepatic C18:0 was also decreased in triheptanoin-fed mice versus dietary controls. **B. Skeletal muscle**. Patterns for fatty acids in muscle were similar in muscle to those seen in liver, but less pronounced. Data in bar graphs were normalized to the median of the WT+chow control group, median scaled to 1, but are presented as averages with standard errors. Symbols denote group differences by ANOVA contrast comparisons of log-transformed data (p≤0.05): asterisk, differs from matching chow control, dagger, differs from matching WT control; double dagger, differs from matching SBO control.

Although the normalization of BHBA by triheptanoin diet may not in itself reflect increased beta-oxidation, metabolomics data of free even-carbon fatty acids suggested that intake of triheptanoin diet influenced fatty acid metabolism. Triheptanoin diet resulted in increased detection of fatty acids shorter than palmitate (myristate, laurate, and caprate); palmitate levels appeared normal, but stearate levels were decreased with triheptanoin. This pattern was most prominent in liver extracts (Figure 14A), but also seen in muscle (Figure 14B). The increased detection of medium-chain, even-carbon length fatty acids suggest that fatty acid oxidation could be enhanced in triheptanoin-fed mice. We would not interpret this necessarily as a direct effect on enzyme components of the fatty acid oxidation pathway. Rather, it may be an indirect result of anaplerosis and resumption of TCA cycle provision of reducing factors for the ETC and ATP production; each cycle of beta-oxidation requires investment of 2 molecules of ATP, and also 2 molecules of CoA, the synthesis of which requires ATP. In this context, we noted that free CoA levels were increased with triheptanoin diet in liver (Figure 10). The shifts in metabolomic signature for fatty acids with triheptanoin diet also seem consistent with the lean phenotype of *Mecp2* KO mice on triheptanoin versus SBO.

**Figure 15:**
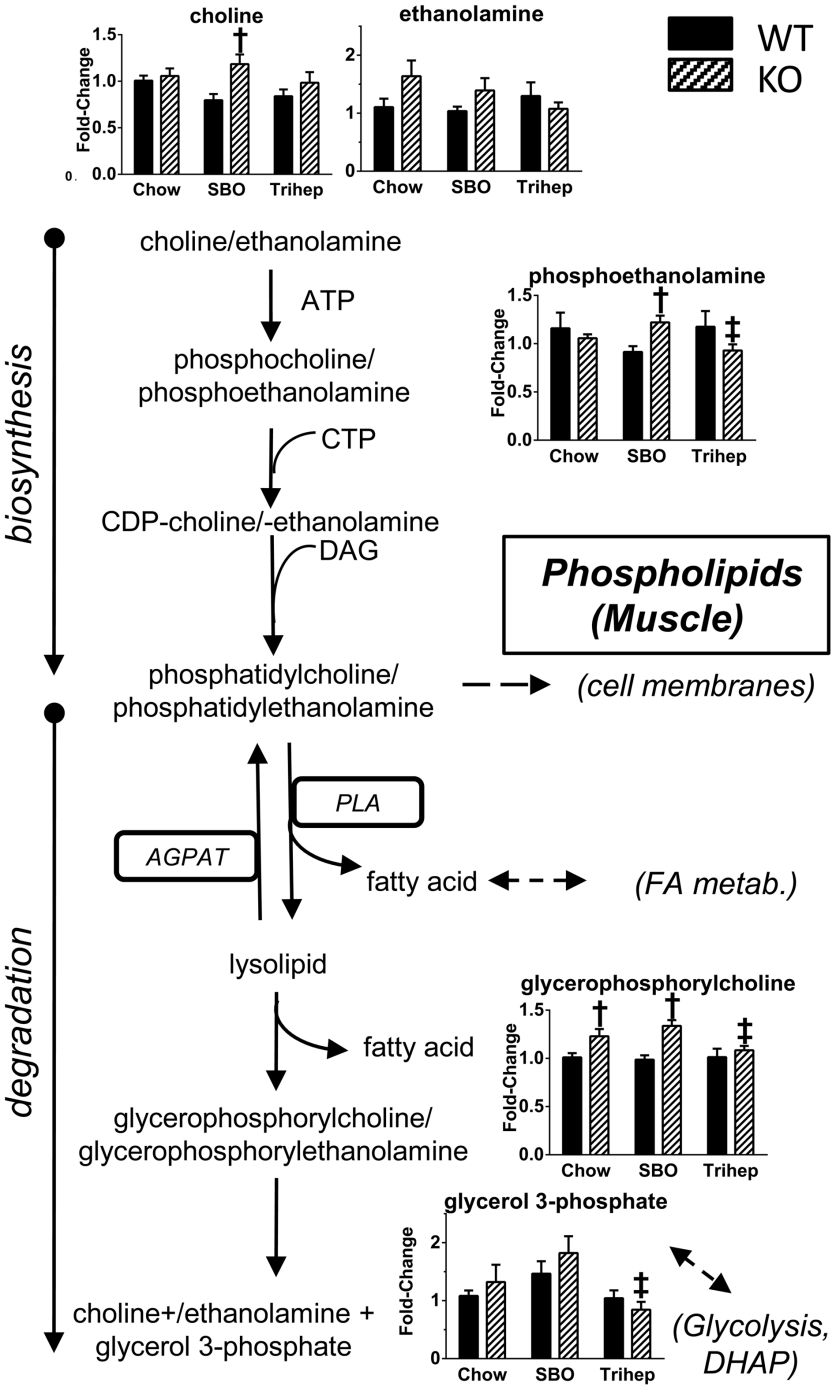
Anaplerotic triheptanoin diet prevents phospholipid degradation in skeletal muscle of *Mecp2* KO mice. The phospholipid degradation product glycerophosphorylcholine was elevated in *Mecp2* KO versus WT on chow or SBO diet. KO mice fed triheptanoin had normal levels of this metabolite. Other detected metabolites are shown. Data in bar graphs were normalized to the median of the WT+chow control group, median scaled to 1, but are presented as averages with standard errors. Symbols denote group differences by ANOVA contrast comparisons of log-transformed data (p≤0.05): asterisk, differs from matching chow control, dagger, differs from matching WT control; double dagger, differs from matching SBO control.

Untargeted metabolomics demonstrated that anaplerotic triheptanoin diet prevented phospholipid degradation in *Mecp2* KO mice. Skeletal muscle of *Mecp2* KO mice on chow or SBO had increased glycerophosphorylcholine, a molecule unique to phospholipid degradation (Figure 15). Triheptanoin diet normalized glycerophosphorylcholine levels to those of WT mice (Figure 15), suggesting that effects of triheptanoin on metabolism can preserve phospholipids and protect the integrity of cellular and organelle membranes. Interestingly, levels of another degradation product, glycerol-3-phosphate, showed a diet-dependent pattern very similar to those of late-glycolytic intermediates (fructose-1,6-bisphosphate through pyruvate, Figure 12), with tendency for higher glycerol-3-phosphate in SBO groups, and suppression with triheptanoin diet. Glycerol-3-phosphate and dihydroxyacetone phosphate of glycolysis are linked metabolically bi-directionally by glycerol-3-phosphate dehydrogenase, with NAD+/NADH as a cofactor. Thus, the pattern of glycerol-3-phosphate normalization with triheptanoin therefore may not reflect decreased phospholipid degradation so much as enhanced glycolytic flux.

## Discussion

In these studies, we investigated the hypothesis that MeCP2-deficient mice could be aided with an anaplerotic diet intervention to improve TCA cycle function. Here, we demonstrate that providing *Mecp2* KO mice with dietary supplement of triheptanoin improves untoward consequences of MeCP2 deficiency. Triheptanoin diet prolonged lifespan, and improved motor coordination and social interaction in male *Mecp2* KO mice. Triheptanoin also improved their overall metabolic profile, preventing adiposity and improving glucose tolerance and insulin sensitivity. Triheptanoin diet greatly normalized the aberrant structural appearance of mitochondria in skeletal muscle of *Mecp2* KO mice. Untargeted metabolomics analyses provided important insights into the metabolic impairments in *Mecp2* KO mice, and supported the hypothesis that anaplerotic diet therapy to enhance TCA cycle flux and fuel oxidation normalizes performance of multiple metabolic pathways to improve metabolic phenotype. The metabolic improvements with triheptanoin also came with functional improvements for RTT-like symptoms in *Mecp2* KO mice. Thus, the results suggest that anaplerotic therapies may aid in therapeutic approaches to RTT and other ASDs.

Our initial characterization of the overall body composition phenotype of *Mecp2* KO mice versus WT suggested some kind of fuel utilization derangement with the absence of MeCP2. Although KO mice were the same weight as WT, they were fatty. We note that other laboratories reported indications of metabolic syndrome, similar to those in our study of *Mecp2* KO mice: male Mecp2-null mice, from crossings of C57BL6 males with heterozygous female *Mecp2* ^TM1.Bird/+^ mice on a 129S6 background, were overweight and obese, exhibited poor responses in GTT and ITT[33], and had increased serum cholesterol and triglycerides[33], [47]. A recent assessment of the preclinical research has suggested that inclusion of 129 genetic background permits this type of metabolic syndrome, whereas pure C57BL6 background results in mutants with lower body weight and no syndrome[48]. While this may be the case for some models at relatively early time points, a recent study of female heterozygous MeCP2 mutants showed that those that had some C57BL6 background but no 129 background were overweight at one year of age; it is not known at what point they became overweight, or if there would have been other indications of metabolic syndrome prior to the one-year time point[49]. Although most RTT patients are not obese, they often have oral and gastrointestinal dysmotilities that should be taken into consideration; obesity does occur in some patients with the “preserved speech” variant of Rett syndrome that has milder symptoms[50]. Thus, overweight or adiposity as a read-out of overall metabolic imbalance may be prevented on a case-by-case basis by other severe symptoms of the disease.

The increased adiposity of our *Mecp2* KO mice coincided with a decrease in lean mass. Hence, *Mecp2* KO mice were not anabolic in regard to skeletal muscle mass, but were instead storing available calories as fat. The adiposity of *Mecp2* KO mice was not due to increased food intake; hence, the animals were not in a positive energy balance from this perspective. Many patients with Rett syndrome have decreased growth and poor muscle mass. Decreased muscle mass could contribute to hypotonia as seen in RTT patients, and although motor impairments in RTT are considered to have a mainly CNS and neuromuscular basis, decreased muscle mass could contribute to the poor performance of *Mecp2 KO* mice on the rotarod. Due to these data, in context with a growing appreciation that mitochondrial dysfunction may play a role in RTT, and because anaplerotic diet therapy has aided a wide range of metabolic diseases, we hypothesized that there may be a deficit in general fuel substrate utilization in *Mecp2* KO, irrespective of specific derangements, that could benefit from anaplerotic therapy using triheptanoin.

Providing triheptanoin diet to male *Mecp2* KO mice extended their lifespan significantly, despite the late developmental age of 4 weeks at which they began treatment. *Mecp2* KO mice on triheptanoin diet also had significantly improved motor function; whereas KO mice on the control SBO diet showed deteriorating rotarod performance over time, the KO mice given triheptanoin diet from 4 weeks of age maintained their level of rotarod performance. Importantly, triheptanoin diet also improved sociability, a CNS-driven function, in the KO mice. It is interesting to speculate that providing an anaplerotic fuel to the dams, and therefore to the pups at postnatal or even prenatal stages, could prevent the lowered motor function seen in *Mecp2* KO mice at 4 weeks of age, or could provide even greater extension of lifespan or improved sociability. Such an approach may model the anticipated treatment intervention in human patients, who typically show symptoms during infancy and early childhood.

Coinciding with the functional improvements from triheptanoin diet therapy in *Mecp2* KO, we also noted that triheptanoin prevented the overall body composition derangement seen in KO on the control diets. Indeed, the SBO control diet, though not obesogenic in WT mice during this study, rapidly increased body weight, adiposity, and serum leptin in the *Mecp2* KO, whereas triheptanoin diet resulted in completely normal outcomes for these measurements. The exaggerated adiposity seen in the KO on SBO supports that MeCP2 deficiency confers a reduced ability to catabolize the additional load of even-carbon-length fatty acids in the SBO diet, exacerbating the underlying metabolic problems in *Mecp2* KO.

*Mecp2* KO mice on normal chow tended to have elevated serum insulin at 7 weeks of age. GTT of *Mecp2* KO mice on chow showed clear glucose intolerance as early as 4 weeks of age, prior to significant increases in adiposity, and ITT results did not show clear insulin insensitivity until 8 weeks of age. This suggests a problem with glucose disposal in peripheral tissues, or impaired glucose utilization, even before there was a serious problem with insulin sensitivity in *Mecp2* KO mice on a normal diet. However, *Mecp2* KO mice contending with additional load of even-chain fatty acids from SBO diet were hyperinsulinemic, and had very poor responses in GTT and ITT, suggesting that the KO mice are prone to develop type-II diabetes that can exacerbate their problems with glucose disposal. In stark contrast, treatment of *Mecp2* KO mice with a triheptanoin diet resulted in completely normal GTT and ITT responses. It is important to point out that this normalization of blood glucose would not necessarily be predicted from published data; triheptanoin is proposed to be gluconeogenic, based on rates-of-appearance calculations in labeled tracer isotopomics studies in anesthetized rats infused intraduodenally for 2-hr with triheptanoin[42], [51], and in studies of perfused liver[52], and in some cases triheptanoin has been shown to increase blood glucose[51]. Nevertheless, the current results with chronic, meal-paced triheptanoin, show normal blood glucose homeostasis. The insulin receptor is sensitized by ATP[53]; if triheptanoin effects on metabolism provide *Mecp2* KO mice with sufficient ATP to allow optimal insulin receptor function, any gluconeogenesis that may result from triheptanoin diet may be balanced with enhanced glucose disposal.

Liver PEPCK is a rate-limiting enzyme for gluconeogenesis, a contributing factor in hyperglycemia of Type-II diabetes[34], in which insulin resistance prevents the normal dominant down-regulation by insulin of PEPCK expression[54]. As hypothesized, *Mecp2* KO mice on the SBO control diet had significantly higher mRNA expression for liver PEPCK than WT mice. However, *Mecp2* KO mice on triheptanoin diet had increased PEPCK mRNA compared to WT, and triheptanoin also increased PEPCK expression in WT versus WT-SBO controls. PEPCK can serve as a gateway to alternative biosynthetic pathways, and even to pyruvate recycling for additional oxidation[27], [55]. A broader, critical role for PEPCK in all tissues is likely to be cataplerosis, the removal of excess TCA cycle anions from the mitochondrial matrix that could occur from anaplerosis[27]. Indeed, it has been shown that liver PEPCK level is highly correlated with TCA cycle activity, with surprisingly low correlation of PEPCK content with gluconeogenic flux[56]. There were no effects of triheptanoin on levels of gluconeogenic intermediates in liver, although this does not speak directly to metabolic flux through the pathway. Although triheptanoin has been shown to be gluconeogenic, the increased PEPCK gene expression in response to triheptanoin diet, regardless of mouse genotype, may serve mainly as cataplerotic balance to the triheptanoin-induced anaplerosis.

Structurally abnormal mitochondria have been reported in skeletal muscle of RTT patients[14]–[18]. Similarly, we show that our *Mecp2* KO mice have swollen mitochondria in skeletal muscle, with disrupted cristae organization. Mitochondrial swelling has been shown in a variety of tissues after cellular stressors including excitotoxicity, exposure to toxic compounds, hypoxia/reperfusion, and oxidative stress[57]–[60]. Swelling results from opening of a mitochondrial permeability transition pore, and causes depolarization of the mitochondrial membrane and disruption of the proton electrochemical gradient needed for ATP production. Swelling also disrupts mitochondrial membrane integrity, which can lead to leakage of mitochondrial contents. Such leakage of TCA cycle intermediates would constitute a non-physiological cataplerosis that could be detrimental to mitochondria if endogenous anaplerotic sources are insufficient. Thus, we expected that anaplerotic diet would help mitochondria function better metabolically by resupplying TCA cycle anions, but we did not necessarily expect that triheptanoin diet would address mitochondrial morphology. However, triheptanoin diet greatly normalized the appearance of skeletal muscle mitochondria in *Mecp2* KO mice. One interpretation of this is that anaplerosis with triheptanoin addresses a metabolic deficit that leads to the mitochondrial swelling in the first place. The deficit may be an impaired ability of mitochondria to oxidize fuel for normal anabolic metabolism, such as to build and maintain cell and organelle components. More investigation is required to assess this and other possibilities.

Decreased levels of mitochondrial enzymes and components of the ETC have been reported previously in RTT. Skeletal muscle biopsies and isolated mitochondria from RTT patients have shown lowered of cytochrome c oxidase, succinate cytochrome c reductase, NADH cytochrome c reductase, and NADH dehydrogenase[14], [15]. Decreased gene expression for NADH dehydrogenase has been reported in brains from *Mecp2* KO mice[22], although other reports with hippocampal tissue did not find clear differential expression of mitochondrial enzymes or ETC components[37]. Here, in addition to abnormal mitochondrial appearance, we also show decreased levels of COX-4 protein in *Mecp2* KO muscle. Importantly, triheptanoin diet significantly increased COX4 protein, again suggesting that addressing an underlying metabolic problem in *Mecp2* KO mice with exogenous anaplerotic substrate could be critically important for aiding mitochondrial metabolism in RTT.

To explore potential biochemical mechanisms by which triheptanoin diet improved the aforementioned parameters in *Mecp2* KO mice, we performed untargeted metabolomics. PCA plots showed clear separation of triheptanoin-fed mice from other groups (Figure 8), and the principal source of difference appeared to be presence of odd-chain fatty acids in livers of mice fed triheptanoin. This was anticipated; during intraduodenal infusion of triheptanoin, very little heptanoate from triheptanoin hydrolysis appears to escape hepatic metabolism[42]. Mice fed triheptanoin were not distinguishable by diet based on PCA score plots from muscle samples, as most dietary heptanoate would not reach muscle. Nevertheless, there was a clear triheptanoin-based distinction of muscle samples based on heat maps, as for liver (Figure 9).

In the liver, *Mecp2* KO mice on the control chow diet had higher steady-state levels of malate and fumarate versus WT controls, but without elevated citrate (Figure 10), indicating that TCA flux toward both OAA replenishment and citrate formation is impaired with MeCP2 deficiency. Triheptanoin diet had an anaplerotic effect for TCA cycle in liver, clearly evident as an increase in steady-state levels of citrate, indicating that OAA replenishment had occurred and acetyl-CoA could enter the cycle.

For muscle, the TCA cycle metabolomics pattern was more subtle. An increase in steady state citrate level was not seen with triheptanoin diet in muscle. This may not be surprising, given that the majority of dietary heptanoate is metabolized by the liver, with only some of the carbons leaving as BKP ketone for anaplerosis in other tissues. Nevertheless, patterns of TCA cycle intermediates in muscle of triheptanoin-fed mice still supported the notion of increased TCA cycle flux. Levels of malate, fumarate, and succinate were all increased in muscle from WT mice on anaplerotic diet, indicating an increased supply of succinyl-CoA (Figure 11). An increase in levels of late-TCA cycle intermediates was not seen in the KO mice, but this does not rule out increased flux through this part of the pathway to replenish OAA.

Another difference between liver and muscle patterns is related to acetyl-CoA, for its potential entry to the TCA cycle as free CoA. In liver, CoA was increased with triheptanoin diet in the same manner as citrate. Our analysis does not distinguish between different subcellular pools of free CoA, but we interpret the CoA increase as mitochondrial, available for pyruvate dehydrogenase to make acetyl-CoA, which then is used with OAA by citrate synthase to make citrate.

For muscle, a distinguishing feature was the striking increase in acetyl-CoA and CoA levels in SBO-fed mice versus either chow or triheptanoin (Figure. 11). This same pattern was also seen for pyruvate and late-glycolytic intermediates in muscle. These increases were seen for both the *Mecp2* KO and WT mice. The interpretation of these observations could be complex, because WT and KO mice clearly had different body compositions indicating different fuel utilization, and the differences become further exaggerated with the SBO diet, since SBO diet worsens adiposity and metabolic biomarkers in KO mice. In these conditions, the levels of late-glycolytic intermediates could be high due to impeded acetyl-CoA entry into the TCA cycle. Fatty acid metabolism and amino acid metabolism could also impact acetyl-CoA levels. For muscle, the important features for interpreting the glycolytic pathway patterns may be the decreases in G-6-P and glucose seen with the triheptanoin diet, accompanied by normal levels of late-glycolytic intermediates (Figure 12). We interpret this as normalized or perhaps more efficient glycolysis, due to efficient entry of acetyl-CoA into the TCA cycle. This interpretation seems likely in context with the improved glucose disposal by *Mecp2* KO mice in the GTT and increase in insulin sensitivity in the ITT.

A defining metabolic feature of *Mecp2* KO mice was increased BHBA in liver and muscle (Figure 13). Elevated ketone levels are often interpreted as increased fatty acid beta-oxidation rate. However, the fatty phenotype of *Mecp2* KO mice does not indicate a state of increased fatty acid oxidation. Even under physiological conditions, ketone bodies are produced at moderate levels[45]. Ordinarily, these ketones are utilized. However, if the processing capacity of the TCA cycle is low due to decreased levels of catalytic intermediates, acetyl-CoA may be directed to ketone synthesis[46]. Anaplerotic triheptanoin diet, which increases TCA cycle flux, normalized BHBA levels in *Mecp2* KO tissues to those seen in WT mice. On the surface, this would seem in contradiction with previous clinical studies in patients with fatty acid oxidation deficit (VLCAD), in whom triheptanoin diet increased C4-ketones modestly[30]; rapid hepatic metabolism of heptanoate to propionyl-CoA and acetyl-CoA is thought to exceed TCA cycle capacity to oxidize acetyl-CoA[26], so the excess acetyl-CoA is channeled to C4-ketones which are exported from liver along with the C5-ketones. However, part of the pathology for patients with fatty acid oxidation defect is an inability to make ketones; thus, an increase in C4-ketones with triheptanoin diet would be easily detected. Our *Mecp2* KO mice had increased tissue BHBA that normalized with triheptanoin diet. It is possible that any C4-ketones produced by liver after triheptanoin diet were utilized by tissues along with the pre-existing high levels of BHBA. One important benefit from lowering tissue BHBA in *Mecp2* KO mice could be relief from BHBA-induced inhibition of insulin-stimulated glucose transport (via insulin-mediated phosphorylation of protein kinase B) in muscle[61]. This would be consistent with the apparent benefits for glycolysis, and for the improved outcomes in GTT and ITT, in *Mecp2* KO mice on triheptanoin diet. How the relative increase of tissue BHBA in *Mecp2* KO mice relates to RTT in humans is not clear. RTT patients are not reported to have ketosis (excess ketone formation), and a classical ketogenic diet can control seizures in RTT patients and improve sociability[62], [63], suggesting that at least in brain the ketones may be utilized. However, triheptanoin is effective in several mouse seizure models [64], [65] and has been suggested as an even more effective treatment for epilepsy [23].

From analysis of free fatty acids by the untargeted metabolomics, it appeared that the triheptanoin diet kept palmitate levels in liver and muscle normal, and decreased stearate, while simultaneously increasing levels of the shorter myristate, laurate, and caprate. This fatty acid shortening suggests more efficient fatty acid oxidation, which seems consistent with the leaner phenotype of KO mice on triheptanoin. We note that this would not necessarily register as a whole-animal increase in fat oxidation (decrease in RER), particularly if glucose oxidation is increased at the same time, as our data suggest. Although VO_2_ and EE were not increased significantly with triheptanoin diet versus SBO, there was a trend in that direction. When the decrease in *Mecp2* KO physical activity is taken into account, which would be expected to decrease VO_2_ and EE, it is plausible that the triheptanoin diet produces fairly substantial increase in basal oxidation rate in the KO mice that counteracts any decrease associated with the low physical activity.

Metabolomics indicated that an anaplerotic triheptanoin diet prevented phospholipid degradation in skeletal muscle of *Mecp2* KO mice; these mice had decreased degradation product glycerophosphorylcholine that was normalized with triheptanoin diet. It is important to consider the possibility that phospholipid degradation could result from MeCP2 deficiency, resulting in non-physiological cataplerosis by leakage of TCA anions from mitochondria, and thus KO mice may require exogenous anaplerotic fuel to maintain energy production, which in turn may help repair membranes. Alternatively, deficits seen with *Mecp2* KO could originate with abnormal ETC and an energy deficit that then stimulates scavenging of fuel substrate from cellular components including phospholipids.

Our results demonstrating the success of a triheptanoin anaplerotic diet in treating defects incurred by MeCP2 deficiency in mice suggests *de facto* that the KO mice have suboptimal mitochondrial TCA cycle processing of nutrient substrates. The physiological and metabolomics analyses support that the male KO mice used in these studies have multiple indications of metabolic dysregulation that affect both carbohydrate and fatty acid metabolism. It remains to be determined whether the mitochondrial deficits arise directly from improper gene expressions of mitochondrial ETC or TCA enzymes that may result from MeCP2 deficiency, as some reports suggest, or if other factors lead to unhealthy mitochondria and a non-physiological cataplerosis that would benefit from the anaplerotic intervention. Further examination of the nature of the metabolic dysfunctions in RTT is important, as is the effect of potential metabolic therapies. Other laboratories have reported *Mecp2* KO mice with fattiness, poor GTT and ITT responses, and elevated serum triglycerides and cholesterol [33], [47], and a mutagenesis suppressor screening produced a line of Mecp2-null mice with defect of squalene epoxidase, a rate-limiting enzyme for cholesterol synthesis, and with significantly diminished RTT-like symptoms[47]. However, the metabolic treatments tried in those studies had very narrow therapeutic windows: low doses of insulin growth-like factor 1 or insulin[33], or statins[47], improved RTT symptoms, but higher doses were detrimental. Human RTT patients have varying degrees of MECP2 deficiency or defect, and a wide range of symptom presentation and severity, which may further complicate the application of therapies clinically. The *Mecp2* KO mice in the present study did not suffer untoward effects with triheptanoin diet; further study is warranted to investigate this issue, but the mitigation of RTT-like symptoms with triheptanoin diet was impressive in the present studies in mice, and has been well-tolerated in patients with other metabolic diseases[24], [28]–[31].

The current work describes metabolic changes in a RTT-like model, and the normalizations of these changes with triheptanoin diet. RTT research has focused mainly on neurons due to observations of altered dendritic morphology and synaptic changes[66]–[68], and genetic deletion studies have shown that neuronal KO of Mecp2 produces severe RTT-like symptoms in mice[69]. Neuronal activity is highly dependent on energy metabolism [70], and RTT affects brain development during the most dynamic phases of growth and establishment of neural connectivity[71]. In this context, brain tissue from a RTT patient has shown structural mitochondrial abnormalities similar to those seen in skeletal muscle[72], and some reports on mouse models of RTT[22], but not all[37], have shown decreased gene expression of mitochondrial ETC enzymes. However, the concept of RTT as a purely neuronal disease is changing, as the potential involvement of astrocytes in RTT pathophysiology becomes more evident[73], [74]. Furthermore, RTT patients show hypotonia[75] and myocardial dysfunction[76] which could arise from local tissue dysfunctions. Importantly, triheptanoin diet improved sociability in the *MeCP2* KO mice; sociability is a CNS-driven function. Although present experiments focused on metabolism in peripheral tissues, the CNS may also receive benefit from anaplerotic therapy; the C5 ketones from hepatic metabolism of heptanoate would be expected to reach the CNS. Thus, our results showing amelioration of RTT-like signs and metabolic disturbances with triheptanoin is an important advance, as this is a potential therapy to treat patients. The present work supports the concept that mitochondrial metabolic imbalance contributes to RTT pathophysiology, for which anaplerotic diet may be an effective and tractable therapeutic approach.

## Materials and Methods

### Ethics Statement

All experimental protocols were approved by the Johns Hopkins University Institutional Animal Care and Use Committee, in accordance with the National Institutes of Health guidelines for care and use of laboratory animals.

### Animals

Male WT and *Mecp2* KO were used for these studies to avoid complications of X-inactivation. Male hemizygous Mecp2-KO mice were produced as described previously for our other work using olfactory system[77], [78]. In brief, female Mecp2 heterozygous mice on an essentially pure BALB/c background were originally provided by Dr. Rudolf Jaenisch[79]. Male mice (gene-targeted for M72-IRES-taulacZ to visualize M72 olfactory receptors) on a 129/SvEv-C57BL/6 background were mated with the female Mecp2-heterozygous mice to generate double mutant females (Mecp2-heterozygous+M72-homozygous). The colony has been maintained on a mixed 129SvEV-C57BL6-BALB/c background. Male mice hemizygous for Mecp2-KO for the studies are produced by crossing heterozygous-Mecp2 females with wild-type males from the colony. Mice were bred and housed in a vivarium (22±1°C, 40±10% humidity, 12 h:12 h light:dark cycle) in ventilated caging (Innovive, Inc.), with 2–5 littermates per tub after weaning at 28 days of age.

### Diets

Maintenance diet was chow (Teklad 2018SX, Harlan Laboratories) until weaning at 28 days of age, after which mice were allowed to continue on the chow, or in further studies were randomly assigned to receive either a diet supplemented with triheptanoin (C7TG, BOC Sciences), or an isocaloric diet enriched with soybean oil (SBO), the fat source in the chow. These test diets were custom-synthesized by Harlan Laboratories (C7TG diet TD.07356, SBO diet TD.07357). Compositions of C7TG and SBO diets are described in Table S1, with comparisons where possible to the chow.

### Social Interaction

Mice were tested at 8 weeks of age, after 4 weeks on test diets, for degree of social interaction in the absence of direct physical contact, in a test modified from published work[80]. A home cage environment was outfitted with a plastic partition that divided one third of the cage from the remaining two thirds. The partition had holes to allow passage of air, but not physical contact, between mice on either side of the partition. The larger portion of the cage housed the test mouse, while a novel WT mouse was placed in the smaller cage portion. The test mouse's cage area has marked as the half nearest the novel mouse and the remaining half farthest from the novel mouse. Time spent in the compartment nearest to the novel mouse was recorded using a timer, and expressed as percentage of the total 10-min test.

### Rotarod

Animals were tested at 4, 5, 6, 7 and 8 weeks of age. At the first time point, the mice performed a habituation trial on day 1 during which they were placed on a rotarod rotating at 4 rpm for 60 s. The following day, each mouse was given two trials, during which the rotarod accelerated from 4 rpm to 40 rpm over a period of ten minutes. The maximum trial length was 10 min, with a 60-min rest period between trials. Latency to fall from the rotarod was recorded. For each time point, results from the two trials were averaged per mouse.

### Indirect calorimetry

*Mecp2* KO and WT control mice at 6 weeks of age were monitored for four days in an open-flow indirect calorimeter (CLAMS, Columbus Instruments) with simultaneous measurements of food intake (food on scales) and physical activity (infrared beam breaks, x-axis). Mice acclimated to the individual chambers for three days, followed by one day of stable data acquisition. Rates of oxygen consumption (VO_2_, ml/kg/hr) and carbon dioxide production (VCO_2_) were measured for each chamber every 24 minutes throughout the study. Respiratory exchange ratio (RER = VCO_2_/VO_2_) was calculated by Oxymax software to estimate relative oxidation of carbohydrate (RER = 1.0) versus fat (RER approaching 0.7), not accounting for protein oxidation. Energy expenditure was calculated as EE = VO_2_×(3.815+(1.232×RER)) and normalized for subject body mass (kcals/kg/hr). Average metabolic values were calculated per subject, for each day, and averaged across subjects for statistical analysis.

### Western Blot

Tissues were collected and lysed in RIPA buffer (Sigma) in the presence of protease and phosphatase inhibitors (Thermo Scientific), and samples were prepared in protein sample buffer (62.5 mM Tris-HCl pH 6.8, 10% glycerol, 2% sodium dodecyl sulfate, 1% β-mercaptoethanol and trace amounts of bromophenol blue), boiled, and stored at -80°C. For gel electrophoresis and western blot analyses, samples were run on 4–15% precast Tris-HCl SDS-polyacrylamide gels (BioRad) and transferred to polyvinylidenedifluoride (PVDF) membranes (BioRad). Blots were successively probed with anti-COX4 or -actin antibodies at 1∶1000 dilutions in TBS containing 3% protease free BSA (Sigma). Blots were visualized using SuperSignal chemiluminescence kits (Pierce Biotechnology) and the images were acquired and quantitated using FluorChem Q (Alpha Innotech).

### Real-time Quantitative RT-PCR

Real time quantitative RT-PCR was performed as previously described [78]. Briefly, tissues were collected and frozen immediately in liquid nitrogen and homogenized using a FastPrep Instrument (MP Biomedicals). Total RNA was extracted with TRIzol reagent (Invitrogen) according to the manufacturer's protocol. cDNA was produced using the TaqMan RT-PCR kit (Invitrogen). Real time PCR was performed on an iCycler (BioRad) by using a reaction mixture with SYBR Green as the fluorescent dye (BioRad), a 1/10 vol of the cDNA preparation as template, and 250 ηM of each primer (sequences available upon request). The cycle used for PCR was as follows: 95°C for 180 s (1 time); 95°C for 30 s, 60°C for 30 s, and 72°C for 30 s (40 times); and 95°C for 60 s (1 time). The fold change in the target gene relative to actin endogenous control gene was determined by: fold change = 2^−Δ(ΔC^_T_^)^ where ΔC_T_ = C_T,target_ - C_T,GAPDH_ and Δ(ΔC_T_) = ΔC_T,KO_-ΔC_T,WT_.

### Glucose and insulin tolerance testing, and other blood chemistry measurements

Glucose tolerance tests (GTT) were performed at 4, 6 and 8 weeks of age. Mice were subjected to 6 h food deprivation, followed by intraperitoneal injection of glucose (1 mg/kg). Clean tail-blood droplets were obtained at 0, 15, 30, 60, 120, and 180 min for measurement of blood glucose with a glucometer (BD Logic, NovaMax strips). Insulin tolerance tests (ITT) were performed at 4, 6 and 8 weeks of age. Mice deprived of food for 2 h were injected with insulin (1 U/kg ip) and glucometer readings were taken from tail blood (0, 15, 30, 45, 60, 90 min). Mice were euthanized for tissue harvesting at the indicated time points, and blood samples were taken for serum measurements of leptin, insulin, adiponectin and/or cytokines by multiplex (Millipore).

### Electron Microscopy

Eight-week-old mice anesthetized with euthasol were perfused intracardially with PBS followed by fixative for electron microscopy (2% glutaraldehyde, 4% paraformaldehyde, 3 mM calcium chloride, 1% sucrose in 0.1 M cacodylate buffer). Fixed muscles were washed in 0.1 M cacodylate buffer (pH 7.2), post-fixed for 2 hours in 2% osmium tetroxide containing 1.6% potassium ferrocyanide, stained *en bloc* with 2% aqueous uranyl acetate (UA), dehydrated through a graded series of ethanol and propylene oxide, embedded in EPON resin and polymerized at 60°C for 24 h. Ultrathin sections were cut at ∼70 nm, stained with 2% UA and lead citrate, and imaged with a 1K AMT camera mounted on a Hitachi H7600 transmission electron microscope (Tokyo, Japan).

### Metabolomics Analysis

Eight-week-old mice were euthanized and samples of liver and skeletal muscle were removed and snap-frozen in liquid nitrogen. Metabolomic profiling was performed by Metabolon as previously described in detail[81], [82].In brief, samples were maintained at -80 °C until processed. A recovery standard was added prior to extraction for quality control. Samples were extracted using aqueous methanol to remove protein and recover small molecules. Extracts were aliquotted for UPLC/MS/MS (positive mode), UPLC/MS/MS (negative mode), GC/MS, and backup. Samples were placed briefly on a concentration evaporator to remove organic solvent, then frozen, dried under vacuum, and prepared for UPLC/MS/MS or GC/MS. For additional quality assurance and control, samples were included that were extracts of a pool created from a small aliquot of all experimental samples. These QC samples and process blanks were spaced evenly among the injections, and experimental samples were randomly distributed throughout the run.

The LC/MS was performed on a Waters ACQUITY ultra-performance liquid chromatography (UPLC), and Thermo-Finnigan linear trap quadrupole (LTQ) mass spectrometer with electrospray ionization (ESI) source and linear ion-trap (LIT) mass analyzer. Dried sample extract was reconstituted in LC-compatible solvents with injection standards at fixed concentrations to ensure injection and chromatographic consistency. One aliquot was analyzed using acidic, positive ion optimized conditions and the other using basic, negative ion optimized conditions in two independent injections using separate dedicated columns. Extracts in acidic conditions were gradient eluted using water and methanol containing 0.1% formic acid, while basic extracts used water/methanol with 6.5 mM ammonium bicarbonate. Samples for GC/MS analysis were re-dried under vacuum desiccation for at least 24 hours prior to derivatization under dried nitrogen using bistrimethyl-silyl-triflouroacetamide (BSTFA). The GC column was 5% phenyl, with temperature ramp from 40° to 300°C in 16 minutes. Samples were analyzed on a Thermo-Finnigan Trace DSQ fast-scanning single-quadrupole mass spectrometer using electron impact ionization.

Raw data was extracted, peak-identified and QC-processed using Metabolon's hardware and software. Compounds were identified by comparison to more than 2400 library entries of purified standards. Missing values were assumed to be below the level of detection. However, biochemicals that were detected in all samples from one or more groups, but not in samples from other groups were assumed to be near the lower limit of detection in the groups in which they were not detected. In this case, the lowest detected level of these biochemicals was imputed for samples in which that biochemical was not detected. Data were normalized to the median of the WT+Chow control group, and scaled with that median set to 1. Statistical analysis of metabolomics data was conducted as described under Statistical Analysis. Pathways were assigned for each metabolite, allowing examination of overrepresented pathways.

### Statistical Analysis

All data presented in bar graphs are the mean±SEM from multiple determinations. Non-metabolomics data was analyzed using Prism 6.0 (Graph Pad). Most non-metabolomics data were analyzed by unpaired t-test, or by two-way ANOVA with either Tukey's or Bonferroni tests for group comparisons as appropriate. Kaplan-Meier survival curves of mice were analyzed with Gehan-Breslow-Wilcoxon test. Metabolomics data were analyzed using R software. Metabolomics group sizes: WT+Chow, n = 9; KO+Chow, n = 9; WT+SBO, n = 8; KO+SBO, n = 7; WT+Trihep, n = 8; KO+Trihep, n-7. Following log transformation of the normalized, median-scaled data (scaled to median of WT+Chow control group), data were analyzed by ANOVA contrasts to identify biochemicals that differed significantly (p≤0.05) between experimental groups. In addition, false discovery rate was estimated by q-value, with q≤0.05 lending higher confidence in the validity of a significant finding. Two-way ANOVA of contrasts identified main effects of diet or genotype, and diet-genotype interactions. Principal Component Analysis graphs were made using R software. Heatmaps were made using Heatmap Builder, version 1.1 (http://ashleylab.stanford.edu/tools/tools-scripts.html). Within heatmaps, each row contains the data from all samples for the identified metabolite. Heat maps are calibrated on a twenty-five point color gradient with highest and lowest metabolite levels as bright red and bright green, respectively. Comparisons are made solely within metabolite. Statistics tests and outcomes are reported in figure legends.

## Supporting Information

Table S1 **Compositions of triheptanoin diet (C7TG) and soybean oil control diet.**
(XLSX)
